# Breaking the mold: Study strategies of students who improve their achievement on introductory biology exams

**DOI:** 10.1371/journal.pone.0287313

**Published:** 2023-07-03

**Authors:** Amanda J. Sebesta, Elena Bray Speth

**Affiliations:** Department of Biology, Saint Louis University, St. Louis, MO, United States of America; Universidad Central de Chile, CHILE

## Abstract

Students’ use of learning strategies (i.e., what students *do* when studying) is linked to their achievement in undergraduate science, technology, engineering, and math (STEM) courses, and several study strategies have been individually associated with course and exam grades in multiple contexts. In this study, we surveyed students in a learner-centered, large-enrollment introductory biology course about their study strategies. We aimed to identify groups of strategies that students often reported together, possibly reflecting broader approaches to studying. Exploratory factor analysis revealed three groups of study strategies frequently co-reported (which we named *housekeeping strategies*, *use of course materials*, and *metacognitive strategies*). These strategy groups map onto a model of learning that associates specific suites of strategies to phases of learning, which correspond to different levels of cognitive and metacognitive engagement. Consistent with previous work, only some study strategies were significantly associated with exam scores: students reporting higher *use of course materials* and of *metacognitive strategies* earned higher scores on the first course exam. Students who improved on the subsequent course exam reported increasing their use of *housekeeping strategies* and of *course materials*. Our findings contribute to a deeper understanding of students’ approaches to studying in introductory college biology and of the relationships between study strategies and achievement. This work may support instructors in adopting intentional classroom practices to foster students’ development as self-regulated learners, able to identify expectations and criteria for success and to implement appropriate and effective study strategies.

## Introduction

Despite the general increase in science, technology, engineering, and math (STEM) graduates over the last decade, many students who enter college to pursue a STEM degree do not complete one [1, 2]. Much of this attrition notoriously occurs in large-enrollment introductory STEM courses, which have been characterized for decades by their high DFW (drop/fail/withdraw) rates—earning them the title of “gateway,” or “gatekeeping,” courses [3, 4].

Why do so many students struggle to persist and succeed in college STEM programs? Of course, there is no simple answer. The whole college learning experience is a complex system in which student outcomes are influenced by a plethora of interdependent variables [5, 6]. One framework for interpreting the complexity of the learning experience is offered by social cognitive theory, which posits that learning occurs in a social context and is influenced by the *environment*, by learners’ *personal factors*, and by their *behaviors* [7, 8]. Personal, behavioral, and environmental factors are not independent of one another, but are interconnected by a network of reciprocal causal interactions [7, 8]. Such an intricate network of causality poses a daunting challenge for education researchers wishing to explain, and possibly work to improve, student outcomes.

Below, we briefly summarize some findings on key *personal*, *environmental*, and *behavioral* factors influencing students’ outcomes in college, particularly in STEM settings, in order to situate our research on college introductory biology within a broader context, and to explain why we ultimately chose to focus on a specific aspect of learner behavior–the *strategies* students implement when studying–in relation to achievement.

### Personal and environmental factors linked to student outcomes

Many *learner-specific variables* have been studied as potential correlates of college readiness and academic success, including demographic variables [9], motivational constructs [6], measures of prior achievement (such as high-school GPA or standardized test scores) [10], and first-year performance [11]. Studies focusing on learners’ demographic variables in undergraduate biology courses identified disparities in course performance and retention linked to first-generation status [12, 13], gender [14], and race and ethnicity [12, 15]. Motivational beliefs such as self-efficacy [15], value and interest in learning a discipline [12, 16], and implicit theories of ability (mindsets [17]) have also been associated with achievement and persistence in STEM. Students’ early performance in introductory science college courses has been associated with overall course outcomes: for example, early exam grades predicted final grades in both introductory biology [18] and introductory chemistry [19] courses, and students’ performance on clicker questions in the first few weeks of the course correlated strongly with final grades in introductory biology [20]. Importantly, influential learner variables frequently intersect and are difficult to disentangle–a student’s prior achievement, college readiness, and mindset, for example, may be tied to one another, reflecting opportunities and resources that are not equally available to all learners. Such differences in learners’ educational experiences, access to educational opportunities, and available supports generate structural and psychological barriers, leading to educational inequalities between student groups [21].

While individual learner variables are generally outside an instructor’s control, instructors and institutions have a great deal of influence on the *learning environment*. Characteristics of the environment that have been linked to student success in STEM include approaches to instruction and assessment [22], class size [23, 24], and academic and social support [25]. Under the most recent impetus for educational reform [26], college biology courses are increasingly implementing learner-centered, evidence-based, interactive pedagogies grounded in educational theory and the learning sciences [27]. An abundance of evidence shows that learner-centered instructional practices support student learning, engagement, and achievement in STEM [22, 28] and often benefit historically underserved students the most [29–31]. Intentionally inclusive classroom environments [32] and alternative grading systems that build in flexibility and opportunities for improvement [33] can also positively influence students’ experiences and outcomes. Nevertheless, the intersection and reciprocal influences of learner and environmental variables increase the difficulty of determining which factors are most relevant for explaining students’ academic outcomes, as illustrated by studies that have explored the relationships between personal and environmental factors and achievement [34–38].

### Behaviors influencing student outcomes: The role of learning strategies

In social cognitive theory, the third broad factor influencing learning is *behavior*, intended as purposeful actions aimed toward achieving goals [7, 8]. In this framework, the process of intentionally and proactively engaging in one’s own learning is referred to as *self-regulated learning* (SRL) [39, 40]. By definition, self-regulated learners set goals, plan and choose study strategies to work toward those goals, monitor their progress, evaluate the effectiveness of their strategies, and adapt their approaches for different tasks [40–42]. A desirable lifelong outcome of education, SRL is a complex developmental process involving cognitive, metacognitive, behavioral, and motivational engagement [43]. Therefore, SRL is not necessarily acquired or automatically developed by learners as they progress through their studies, but can and should be deliberately coached in educational settings [41, 44–46]. SRL has been a major area of study for decades, resulting in multiple conceptual models grounded in different theoretical frameworks, but all generally converging on the critical role of learners’ agency and behavior in the pursuit of learning goals [8, 43]. Seminal work on SRL, drawing from social cognitive theory, identified a suite of *self-regulated learning strategies*: deliberate, purposeful, and goal-oriented behaviors including goal-setting, environmental structuring, setting consequences for oneself, self-evaluating, organizing and transforming study materials, seeking information, rehearsing and memorizing, seeking social assistance, and reviewing course materials [47].

SRL involves a whole suite of processes integrating cognitive, metacognitive, behavioral, and motivational dimensions [43]; therefore, *learning strategies* or *study strategies* (what students do to learn) represent just one element of this broader phenomenon. Yet, from a practical perspective, study strategies can represent crucial, actionable entry points for classroom conversations and interventions that ultimately aim to foster the development of self-regulation and improve academic outcomes. Strategies can be modeled, taught, and recommended in the classroom to answer concretely the perennial student question, “how should I study for this course?”

Much educational and cognitive psychology research studies, therefore, have focused directly on study strategies in relation to academic achievement [48–50]. Empirical evidence shows that students who frequently implement certain study strategies tend to have higher academic achievement, compared to students who implement these strategies less frequently [48, 51–55]. While some foundational strategies may be generally beneficial across settings, the *effectiveness* of most strategies is dependent, among other things, on the learner’s understanding of the tasks and assessment expectations [56, 57], and on their ability to productively apply strategies aligned with the cognitive demands of the tasks [58, 59]. In college STEM courses that emphasize concept application and analysis of novel problems, for instance, students who have little or no experience with these higher-order cognitive tasks may struggle to adapt and meet course demands because they lack familiarity and practice with appropriate cognitive and metacognitive strategies [60]. Strategies like record-keeping and memorizing/rehearsing (*surface learning* strategies aimed at building and consolidating factual knowledge) may be effective and sufficient when learners are expected to simply reproduce information [61, 62]. Conversely, if students are expected to apply and integrate knowledge, they may benefit from adopting *deep learning* strategies such as elaboration, concept-mapping, evaluation and reflection, and collaborative learning [61–64].

### Applying learning strategies in context

Contextual cues, including perceived complexity of the task at hand, format and cognitive demands of assessment, instructor and peer advice, can influence students’ *choice* of strategies [60, 65–70]. However, students also tend to adopt strategies based on their preferences, experiences, and other individual variables such as prior knowledge, attitudes, and goals [59, 60, 71]. Ideally, college learners would be able to identify and understand assessment demands and task expectations in each learning environment, and select appropriate and effective study strategies (a hallmark of SRL). Not all students, however, come to college already able to self-regulate their learning, equipped with a broad suite of strategies and able to determine which strategies are most productive in the context of each course, discipline, or task [72–75]. Students can learn how to apply strategies that support learning and achievement through direct instruction and practice embedded within courses, as well as through extracurricular interventions [52, 76–79]. There is evidence that, at least at the college level, study skills interventions are most effective when situated within specific course contexts [79].

In meta-synthesis of multiple studies relating study strategies to learning and achievement, authors Hattie and Donoghue to generate a model of learning [59], grounded in the assumption that effectiveness of any strategy depends–for each individual student–on a host of factors, including the student’s existing subject knowledge and their motivation and dispositions, but also the clarity of criteria for course or task success and the student’s understanding of these criteria [59]. Hattie and Donoghue’s model associates different learning strategies with *phases* of learning involving different levels of cognitive and metacognitive engagement. In the *surface acquisition and consolidation* phases, learners build foundational content knowledge, such as facts, concepts, and vocabulary. In the *deep acquisition and consolidation* phases, learners connect ideas and engage with problem solving, concept application, elaboration, and metacognition. The last phase, *transfer*, refers to the learner applying knowledge and skills to inquire and reason about problems in novel contexts and situations. Since these distinct phases have different goals, Hattie and Donoghue posited that not all learning strategies are equally effective across all phases, and that different groups of strategies best support each phase [58, 59, 61]. The phases of learning described in the model are also not necessarily viewed as sequential, discrete steps. They all serve different purposes, and they could occur in a different order or even simultaneously for a learner, depending on that learner’s background knowledge in a discipline and on course context and assessment demands [61]. For example, learners who have had little to no previous exposure to disciplinary content may need to acquire and consolidate baseline knowledge before they can engage with the material on a deeper level. In certain contexts (such as problem-based learning), however, students may need to engage in surface and deep learning simultaneously, thus needing to draw from multiple pools of strategies [58, 59].

### Study aims

In our previous work, we explored students’ self-reported use of learning strategies when studying for exams in a large-enrollment introductory biology course [53]. We used the suite of SRL strategies developed by Zimmerman and Martinez-Pons [47] to create a survey, which we used to identify what strategies our students reported using when preparing for exams, and which strategies were associated with students’ self-reported exam grades [53]. Students who reported earning higher grades on the first and second exams, and students whose grades improved from the first to second exam, reported in aggregate higher use of specific strategies, including self-evaluation, seeking information, and using practice exams. We were, however, limited in our ability to dissect the specific relationship between exam improvement and change in strategy use, because we had surveyed students anonymously and could not link the survey data to achievement data [53].

In the present study, we continue to explore students’ learning strategies in relation to exam achievement in an introductory biology course. Our overarching goal is to better understand what suites of strategies are most effective in our course context, so we can deliberately target these strategies in classroom instruction and individual student advising. Compared to our previous work, we modified data collection protocols to link individual students’ responses on multiple surveys to their actual (not self-reported) exam scores, thus increasing methodological accuracy. Based on Hattie and Donoghue’s proposed model of learning [58, 59] and on the results of our previous study, we anticipated that our students would report adopting multiple strategies concurrently, and we hypothesized that strategies frequently reported together would be related to one another, possibly reflecting different levels of cognitive and metacognitive engagement with the material. Furthermore, we hypothesized that improvement in exam performance over time may be associated with changes in the frequency with which students used groups of related strategies. This research ultimately aimed to:

1. identify patterns of strategy use (suites of co-reported study strategies) that may represent broader approaches to studying, and therefore potentially inform us of students’ level of cognitive and metacognitive engagement with the material in our course context; and
2. investigate whether any patterns of strategy use are associated with achievement on the first course exam, and with exam grade improvement over time.

## Methods

### Learning environment: Course context and student population

#### Course content, assessment, and grading

This study was conducted in a first-semester, large-enrollment introductory biology course for life science majors and premedical/pre-professional health students at a large, private research university in the Midwestern U.S. Course content included principles of cellular and molecular biology, cellular metabolism, genetics, and animal form and function. Assessment included four high-stakes exams evenly spaced throughout the semester (three unit exams and one cumulative final exam), which included a variety of item formats (multiple choice, short answer, and conceptual model building) that targeted the knowledge, comprehension, application, and analysis levels of Bloom’s taxonomy [76]. Exams 1, 2, and 3 were scored out of 50 points whereas exam 4 (cumulative final) was scored out of 80 points. Each exam contributed 15% to the final course grade; homework and in-class assignments together accounted for 15% of the course grade while the laboratory component accounted for the remaining 25%.

#### Instructional approach and student supports

Students prepared for each class period by completing readings, watching screencasts (recorded mini lectures introducing basic facts and concepts), and completing low-stakes homework assignments (similar to a flipped classroom model [80]). In class, students worked in permanent collaborative groups and applied their knowledge to solve problems in discussions, case studies, clicker questions, and worksheets. Student construction, evaluation, and use of conceptual models were also a large component of the instructional approach (described in [81]). Undergraduate learning assistants (LAs) supported in-class activities, with approximately one LA per 35–40 students. At the beginning of the semester, instructors provided guidelines and recommendations on how to study for the course. The key messages offered to students were: to study regularly the pre-class screencasts and readings and complete pre-class quizzes; to attend class and be actively engaged with class work; to use out-of-class supports such as office hours, LA study sessions, and supplemental instruction; to critically review all graded assignments and consider them as study materials; to process information and feedback in a timely manner and do not rely on cramming before exams. LAs reinforced this advice throughout the semester in their conversations with students who attended their study sessions. A week or two before each exam, instructors spent some class time reminding students of the resources at their disposal, reiterating the value of studying in a timely manner and of using course materials such as notes, annotated class slides, instructor screencasts, in-class worksheets, and any graded assignments with feedback. Additionally, instructors made question sets from previous years’ exams available and encouraged students to use them as a form of practice.

#### Student participants

Study participants (n = 427) were students enrolled in three sections of the introductory biology course described above. Most were first-year students majoring in STEM; aggregate demographic descriptors of the student population, based on self-reported data collected in week 3 of the course, are summarized in the supplemental material (S1 Table). Two instructors taught the three sections (one instructor taught two sections, and the other instructor one section). The instructors collaborated closely to develop common instruction and assessment; therefore, students had access to the same course materials, completed the same assignments, and took the same exams.

This work was determined to be exempt by the Saint Louis University IRB (protocol #22988) because it was conducted in the context of a typical educational setting; participants’ written or verbal informed consent, therefore, was not required. Students were informed about the research through an IRB-approved recruitment statement, distributed as a digital copy at the beginning of the semester through the learning management system. The recruitment statement explained participants’ rights and responsibilities, including the right to opt out of the research study. Students confirmed that they had read the recruitment statement by checking a box in the learning management system. We anticipated we would remove from the dataset all students who opted out, but no student declined participation.

### SRL surveys: Learning strategies used to prepare for exams

To assess students’ use of learning strategies, we administered a survey in which students reported how frequently they used each of 17 strategies while studying for an exam. We used the survey from our previous work [53], based on work by Zimmerman and Martinez-Pons [47], with minor modifications to various items (see S1 File for the complete survey, with notes and rationale for specific changes). Each strategy item was followed by a 5-point Likert scale for frequency of use: 1 = *Never*, 2 = *Rarely*, 3 = *Sometimes*, 4 = *Often*, 5 = *Very often*. Students took the SRL survey twice: after receiving their graded exam 1 (SRL1, n = 345) and after receiving their graded exam 2 (SRL2, n = 374). Surveys were administered by a graduate assistant (AJS) through Qualtrics®. Responses were recorded with identifying information, which was eventually replaced by a unique alphanumeric code for each student, to link a single student’s responses from multiple surveys and their exam score data.

### Data analysis

For each of the SRL1 and SRL2 surveys, there were no instances of missing data. We collected instructor-recorded scores for each of the four course exams and converted them into percent scores to facilitate comparison. Students who were missing data for their exam 1 score, or missing data for at least two of the three scores for exams 2, 3, and 4, were excluded from analysis (n = 17). All statistical analyses were performed in R [82], with packages and associated functions referenced below as appropriate. The R scripts for all analyses are available through the first author’s (AJS) GitHub site (https://github.com/ajsebesta/SRLStrategies_DataAnalysisFiles).

To determine if we could pool students with different instructors into a single large sample, we compared (a) reported strategy use on each of the post-exam surveys (SRL1 and SRL2) and (b) exam percent scores between students with one instructor versus the other. We conducted contingency tests (chi-squared tests of independence) to determine whether students’ reported strategy use depended on which instructor they had (instructor 1 vs. instructor 2). For the strategy items, we combined Likert-scale responses “Often” and “Very often” into a “higher use” category and responses “Sometimes,” “Rarely,” and “Never” into a “lower use” category. For each 2x2 contingency test, we applied a Yates’ correction for continuity, and to determine significant differences across multiple tests, we applied a Bonferroni correction (adjusted α = 0.00294 for 17 total tests). There were no significant differences in strategy use between students with different instructors on either survey (see R script, through GitHub link, for statistical results and interpretation). Both groups of students had non-normal distributions for their exam scores, based on Shapiro-Wilk tests (Instructor 1: Exam 1 *W* = 0.95589, *p* < 0.0001; Exam 2 *W* = 0.97027, *p* < 0.0001; Exam 3 *W* = 0.95359, *p* < 0.0001; Exam 4 *W* = 0.95361, *p* < 0.0001; Instructor 2: Exam 1 *W* = 0.90443, *p* < 0.0001; Exam 2 *W* = 0.95458, *p* < 0.001; Exam 3 *W* = 0.9522, *p* < 0.0001; Exam 4 *W* = 0.9374, *p* < 0.0001). A two-tailed Mann-Whitney U test for each of the four exams did not detect any significant differences in exam percent scores based on instructor (Exam 1: *U* = 18751, *p* = 0.6871; Exam 2: *U* = 18738, *p* = 0.6793; Exam 3: *U* = 19432, *p* = 0.5784; Exam 4: *U* = 17099, *p* = 0.1054; Bonferroni correction for multiple analyses applied, α = 0.0125). Accordingly, we combined students into a single sample. The full dataset is provided as supplemental material (S2 File).

We conducted exploratory factor analysis (EFA) to reduce the strategies dataset into a smaller set of variables, for identifying overarching *patterns* of strategy use (factors) that could characterize students’ approaches to studying in our course. We used the responses to the SRL1 survey (n = 345) to identify a suitable factor structure because students had not seen the survey items previously. Based on recommendations for best practices [83, 84], we approached the EFA as an iterative process, beginning with all 17 strategies and successively removing any strategies with weak relationships and little contribution to a robust factor structure. If factor structure was still not satisfactory after removing weakly related variables, we would identify and remove both univariate and multivariate outliers to provide resolution to the factor loadings in another round of EFA. Outlier removal was used as a last resort to preserve as much of the dataset as possible since we were close to the suggested minimum sample size of 300 for conducting EFA [84]. We referenced R script from Zygmont and Smith [85] for outlier detection, which includes the “mult.norm” function from the *QuantPsyc* package [86] for multivariate outliers and the “outlier” function from the *outliers* package [87] for univariate outliers.

Since all 17 strategies had non-normal distributions (Shapiro-Wilk tests; see R script), we used principal axis factoring extraction to reduce the dataset into factors [83, 84]. We chose oblique rotation because we expected that the factors resulting from the EFA would not necessarily have distinct boundaries [83, 84]; this choice reflects our hypothesis that students may use multiple strategies and groups of strategies simultaneously. We applied parallel analysis and a scree test to determine the number of factors that would best fit the data, using the “fa.parallel” function in the *psych* package [88]. The function “fa” from the *psych* package was used to conduct the full exploratory factor analysis. We established a relaxed factor loading cutoff of 0.35 for resolving each strategy’s loading onto a single factor for two reasons: (a) we accounted for measurement error in our survey items, given that the framework we used for developing our survey [79] was different than the reference framework for strategy groupings [48], and (b) we did not intentionally build our survey as a validated measure of SRL [83, 84, 89]. To attribute scores for each resulting factor to a student, we calculated simple sums of the ordinal responses to the associated strategies because this scoring approach is intuitive and preserves variation in the original data [90].

We examined whether any pre-existing differences in students’ reported use of strategies on the SRL1 survey could explain differences in their initial exam performance (n = 345). To determine if higher exam 1 performance (defined by z-score groupings; see next paragraph) was associated with higher (vs. lower) use of any of the individual 17 study strategies, we performed contingency analyses using χ^2^ tests of independence and applied a Bonferroni correction for multiple tests (adjusted α = 0.002941 for 17 total tests). We also conducted Kruskal-Wallis H tests, with post-hoc Mann-Whitney U tests (two-tailed), to determine whether students with different exam scores significantly differed in their scores for any strategy factors that emerged from the EFA (Bonferroni correction for multiple Kruskal-Wallis H tests, adjusted α = 0.0167 for three total tests (see Results section for EFA findings); Bonferroni correction for each set of post-hoc Mann-Whitney U tests, α = 0.0083 for six total pairwise tests).

To determine whether students’ initial exam score predicted their scores on subsequent exams, we performed a linear regression, with students’ exam 1 percent score predicting the average percent score of their exams 2, 3, and 4 (n = 410). To account for differences in exam content and difficulty, we standardized percent scores into z-scores for each exam, and we defined four achievement groups based on z-score distribution: Group 1, below the mean by at least one standard deviation; Group 2, within one standard deviation below the mean; Group 3, within one standard deviation above the mean; and Group 4, above the mean by at least one standard deviation (see S1 Fig for a visual schematic). Table 1 shows the distribution of students across z-score groups on each exam, with exam percent score means, standard errors, and percent score range.

**Table 1:** Exam percent scores (% mean ± standard error, and range) for the entire sample and for students grouped by their z-scores on the exams.

	All students	Group 1	Group 2	Group 3	Group 4
**Exam 1**	77.68 ± 0.69	54.03 ± 0.85	70.52 ± 0.38	84.80 ± 0.29	94.19 ± 0.25
	(n = 410)	(n = 70)	(n = 103)	(n = 162)	(n = 75)
	*range*:	36–62%	64–76%	78–90%	92–100%
**Exam 2**	73.89 ± 0.79	48.00 ± 0.97	65.06 ± 0.39	81.91 ± 0.41	93.87 ± 0.35
	(n = 410)	(n = 62)	(n = 130)	(n = 134)	(n = 84)
	*range*:	26–56%	58–73%	74–89%	90–100%
**Exam 3**	73.11 ± 0.76	48.39 ± 0.83	65.93 ± 0.40	82.24 ± 0.36	92.69 ± 0.34
	(n = 406)	(n = 72)	(n = 117)	(n = 156)	(n = 61)
	*range*:	28–56%	58–72%	74–88%	90–100%
**Exam 4**	74.73 ± 0.77	50.51 ± 0.83	66.64 ± 0.41	83.34 ± 0.37	93.65 ± 0.26
	(n = 407)	(n = 76)	(n = 105)	(n = 154)	(n = 72)
	*range*:	25–58.75%	60–73.75%	75–90%	91.25–98.75%
**Exams 2/3/4 avg.**	73.85 ± 0.73	50.43 ± 0.77	66.32 ± 0.41	81.76 ± 0.36	91.93 ± 0.28
	(n = 410)	(n = 72)	(n = 116)	(n = 143)	(n = 79)
	*range*:	31.5–58.83%	59.08–73.75%	74–88.58%	88.67–98.5%

By binning students into four standardized achievement groups on each exam, we could track each student’s performance over time and characterize it in terms of change from their baseline performance on exam 1. We considered students to have improved their performance if they moved up by at least one z-score group on the next exam. Students who did not improve either maintained similar performance by remaining in the same z-score group, or decreased performance by moving down at least one z-score group. In terms of timing of improvement, we refer to students’ change from exam 1 as:

- *short-term change*, for students who improved from exam 1 to exam 2 but then returned to baseline (or lower);
- *long-term change*, for students who improved from exam 1 to exam 2 and maintained their improvement through the end of the semester; or
- *late change*, for students whose performance improved after exam 2 (on exams 3 or 4).

We pooled all students from Groups 1, 2, and 3 on exam 1 (n = 258) because of their potential for improvement in exam performance, and we conducted a set of two-tailed Wilcoxon signed-rank tests to detect any significant changes in strategy factor scores from the SRL1 to SRL2 surveys. To identify whether any significant changes in strategy use were unique to students who improved on exam 2, a set of tests was done for three different student groups: all students with the potential to improve on exam 2 (n = 258), students who improved from exam 1 to exam 2 (n = 67), and students who did not improve from exam 1 to exam 2 (n = 191). We applied a Bonferroni correction for conducting three total tests for each group of students (based on three factors from the EFA results; adjusted α = 0.0167).

## Results

### Exam 1 score predicts subsequent course exam scores

**Fig 1:**
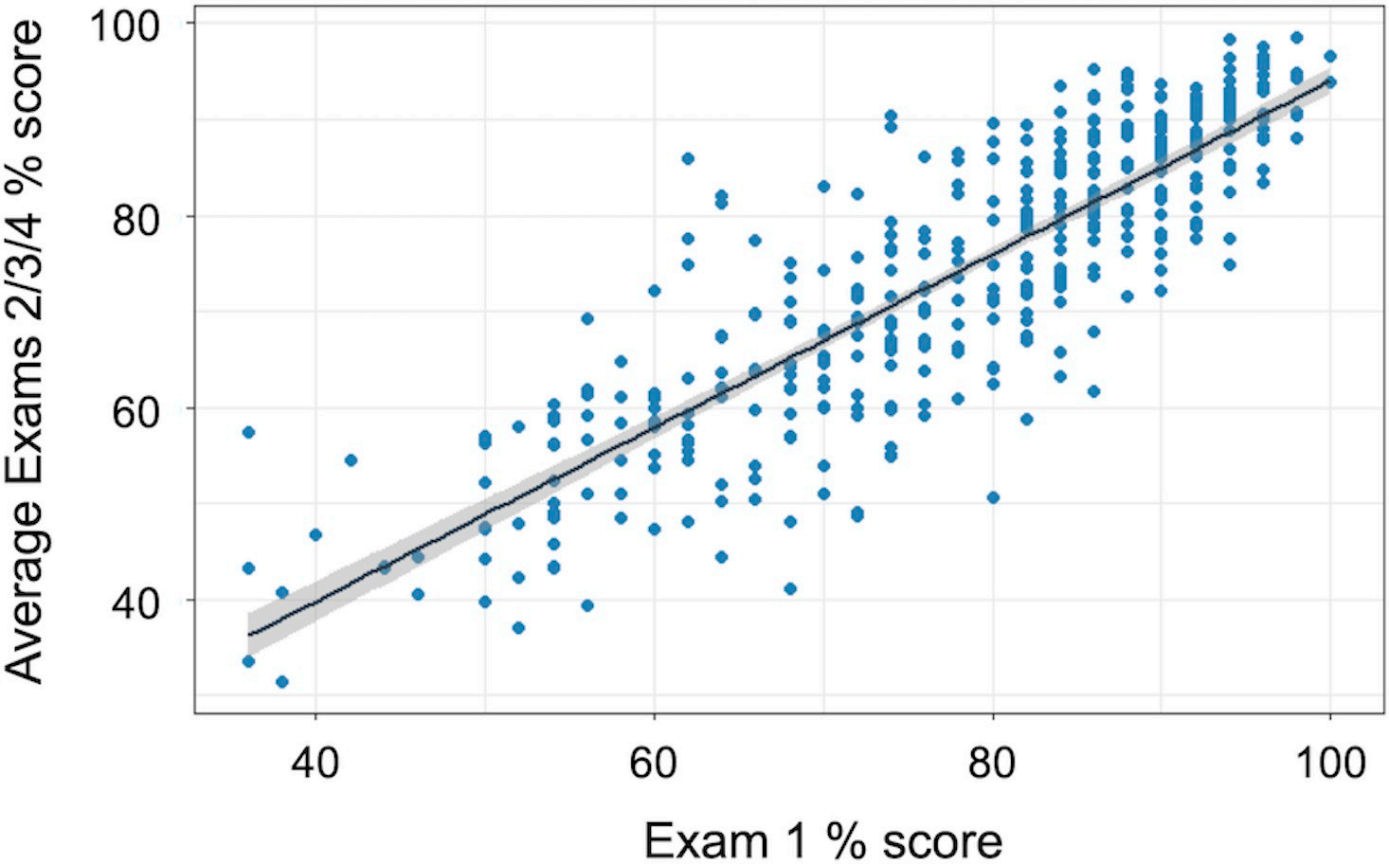
Exam 1 score strongly and significantly predicts subsequent exam scores (defined as the average percent score of Exams 2, 3, and 4) and explains approximately 74% of the variance (R^2^ = 0.737). The 95% confidence interval for the regression line is also shown.

We satisfied the following assumptions for a univariate linear regression: the exam percent scores were significantly correlated with one another (Spearman’s rho = 0.855, *S* = 1660336, *p* < 0.0001), the model residuals were normally distributed (Shapiro-Wilk test: *W* = 0.99295, *p* = 0.0514), and model residuals had a mean of zero. Although neither variable was normally distributed (Shapiro-Wilk tests, Exam 1% score: *W* = 0.94209, *p* < 0.0001; Average Exams 2/3/4% score: *W* = 0.95903, *p* < 0.0001), they had nearly identical distribution shapes that permitted fair comparison. Based on our linear regression, students’ exam 1 percent score strongly and positively predicted the mean percent score of their subsequent three exams (n = 410; *F*(1, 408) = 1142, *p* < 0.0001; Fig 1). Exam 1 explained approximately 74% of the variance in exams 2/3/4 (R^2^ = 0.737); the regression equation is: *Average exams 2/3/4% score* = 3.701 + 0.903(*exam 1% score*).

We also tested whether exam 1 predicted subsequent exam scores for three additional student cohorts in the same course, taught by the same instructors in consecutive academic years. We found that exam 1 percent score strongly and positively predicted the average exams 2/3/4 percent score for all three additional cohorts (linear regressions; S3 File and S2 Fig; data provided in S4 File).

### SRL strategies associated with Exam 1 score

Six SRL strategies were significantly associated with exam 1 score (χ^2^ tests of independence): self-evaluation, reviewing graded work, monitoring understanding, seeking instructor assistance, using practice exams, and reviewing the textbook/screencasts (S2 Table). Students who earned higher exam scores (Group 4, above the mean by > 1 s.d.) reported higher use of these strategies compared to students with lower exam scores (Group 1, below the mean by > 1 s.d.).

### Exploratory factor analysis: Three study strategy groupings

We first applied EFA to the full SRL1 survey dataset, which included all 345 students and all 17 strategies. We also generated a correlation matrix with all 17 strategies, using Spearman’s rho, as a reference to support our interpretation of the EFA (all strategies had non-normal distributions according to Shapiro-Wilk tests and were on an ordinal scale; see R script for test results). The resulting structure produced five factors, two of which only included two strategies. A factor should have at least three variables load onto it to be considered robust [83, 84], so we deemed the five-factor solution sub-optimal. Testing structure with fewer factors did not provide resolution, so we examined the correlation matrix to identify any strategies with a poor pattern of correlation with the others. We identified and removed four strategies since they had a high number of low correlation coefficients (i.e., at least three-fourths of the possible correlation coefficients, or 12 out of 16, were lower than 0.20): *self-consequating*, *studying with peers*, *seeking peer assistance*, and *seeking other assistance*.

A new round of EFA with the 13 remaining strategies yielded a five-factor structure as the best fit. However, some strategies either failed to load onto a factor or cross-loaded onto more than one factor. Testing structure with fewer factors still failed to provide adequate resolution, so we proceeded to eliminate outliers. We removed from the dataset five students who were identified as (a) both univariate and multivariate outliers simultaneously, or (b) severe univariate outliers (i.e., a student was an outlier for at least three individual strategies). Following outlier removal, EFA yielded a suitable four-factor structure and no cross-loading strategies. One of the factors, accounting for 18% of the variance, had only two strategies load onto it: *seeking information* (factor loading = 0.44) and *keeping records* (0.59). We excluded this factor from further analysis, as well as the strategy *environmental structuring*, which failed to load onto any factor. The final structure included three factors, which we named based on the commonalities among strategies that loaded onto their respective factors (Table 2). ***Housekeeping strategies*** accounted for 20% of the variance and included *organizing and transforming* (0.44), *goal-setting and planning* (0.36), and *rehearsing and memorizing* (0.47). ***Use of course materials*** included the strategies *reviewing notes* (0.64), *using practice exams* (0.64), and *reviewing the textbook/screencasts* (0.54), and accounted for 31% of the variance. The final factor, ***metacognitive strategies***, included *self-evaluation* (0.40), *reviewing graded work* (0.64), *monitoring understanding* (0.60), and *seeking instructor assistance* (0.37), and accounted for 31% of the variance. Given our SRL survey Likert scale (1–5), *housekeeping strategies* and *using course materials* had a score range of 3–15 whereas *metacognitive strategies* had a score range of 4–20 (Table 2).

**Table 2:** The three-factor structure from exploratory factor analysis on the SRL1 survey responses (n = 340).

1. Housekeeping strategies (0.20)	2. Use of course materials (0.31)	3. Metacognitive strategies (0.31)
Organizing/transforming (0.44)	Reviewing notes (0.64)	Self-evaluation (0.40)
Goal setting/planning (0.36)	Using practice exams (0.64)	Reviewing graded work (0.64)
Rehearsing/memorizing (0.47)	Reviewing textbook/screencasts (0.54)	Monitoring understanding (0.60)
		Seeking instructor assistance (0.37)
*Score range*: 3–15	*Score range*: 3–15	*Score range*: 4–20

Each factor is listed with the proportion of variance for which it accounts. Individual strategies under each factor are listed with their respective factor loadings (factor loading cutoff = 0.35).

**Fig 2:**
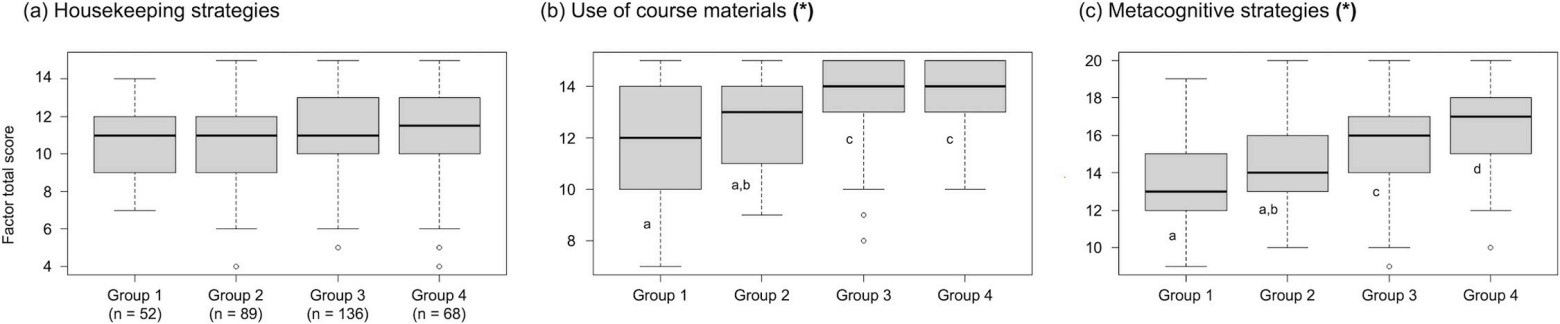
Students with higher exam 1 scores reported higher factor scores for use of course materials and metacognitive strategies, but not for housekeeping strategies, compared to students with lower exam 1 scores (n = 345). Median scores for (a) housekeeping strategies, (b) use of course materials, and (c) metacognitive strategies by exam 1 z-score group. An asterisk in parentheses indicates a statistically significant difference (Kruskal-Wallis H test); lettered superscripts show which groups significantly differ from one another (post-hoc Mann-Whitney U tests; see S3 Table).

Based on Kruskal-Wallis H tests and post-hoc Mann-Whitney U tests, we found that students with higher exam 1 scores (Group 4) reported engaging more frequently, compared to students with lower exam 1 scores (Group 1), with *use of course materials* (*H*(3) = 39.504, *p* < 0.0001) and *metacognitive strategies* (*H*(3) = 65.529, *p* < 0.0001; Fig 2; see S3 Table for pairwise results). However, students with differing exam 1 scores did not significantly differ in their use of *housekeeping strategies* (*H*(3) = 8.2669, *p* = 0.04081; see S3 Table for pairwise results). The results obtained with the factors (strategy groups) were consistent with those of the contingency analyses performed on individual strategies: component strategies of two factors, *use of course materials* and *metacognitive strategies*, were significantly associated with exam 1 performance (S2 Table).

We conducted a post-hoc multiple regression analysis to determine whether these strategy patterns could uniquely explain variance in exam 1 scores, when included as variables in a single analysis. We used generalized linear modeling (GLM) since we could not satisfy the assumptions for a multiple linear regression (see R script), and we used students’ percent scores to facilitate interpretability of the coefficients. Both *metacognitive strategies* and *use of course materials* positively and significantly explained exam 1 percent score, whereas *housekeeping strategies* negatively and significantly explained exam scores: *Exam 1% score* = 35.03–1.07(*housekeeping strategies*) + 1.84(*use of course materials*) + 2.08(*metacognitive strategies*); pseudo-R^2^ = 0.25; AIC = 2680.6. To check for potential redundancy, we tested a GLM with only *use of course materials* and *metacognitive strategies* since these factors emerged as significant from the Kruskal-Wallis H tests. Both factors still positively explained exam 1 percent score, but this two-variable model accounted for less information compared to the three-variable model, based on the change in AIC: *Exam 1% score* = 32.56 + 1.52(*use of course materials*) + 1.74(*metacognitive strategies*); pseudo-R^2^ = 0.23; ΔAIC = 8.3. Thus, the model with all three factors is a better fit than the model with only two factors.

### Exam performance trajectories

**Fig 3:**
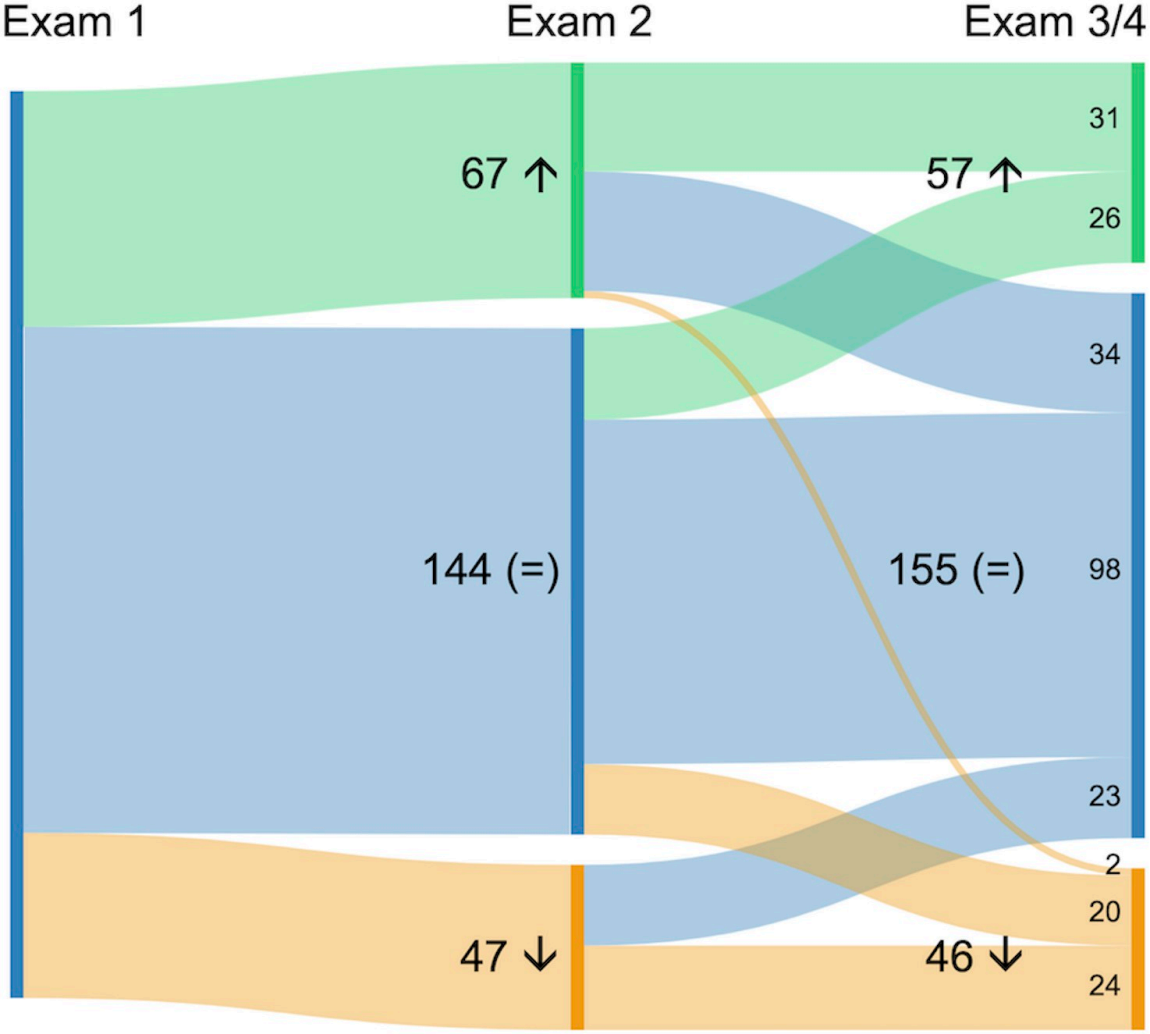
Sankey diagram representing exam performance trajectories across the semester, for students in Groups 1, 2, or 3 on exam 1 with complete data across the SRL surveys (in aggregate, n = 258). Trajectories are shown with exam 1, exam 2, and the average of exams 3/4 as points of interest. From exam 1 to exam 2, improvement is shown in green (moving up at least one z-score group), maintaining similar performance in blue (remaining in same z-score group), and decreasing performance in orange (moving down at least one z-score group). From exam 2 to the average of exams 3/4, students who either maintained their improvement or improved on exams 3/4 are shown in green, those who maintained similar performance or returned to their baseline performance by exams 3/4 are shown in blue, and those who maintained lower performance or decreased on exams 3/4 are shown in orange. The thickness of the lines is proportional to the number of students on each trajectory.

To analyze patterns of improvement over the course of the semester, in relation to self-reported use of study strategies, we focused on the subset of students who had completed both SRL surveys and had the most room for improving upon their exam 1 scores (z-score Groups 1, 2, and 3; n = 258). Students who earned the highest scores on exam 1 (Group 4; n = 67) were excluded from analysis since they had little room for improvement. We found that most students within the subset of interest performed consistently on course exams throughout the semester (i.e., remained in the same z-score group as on exam 1; n = 98; Fig 3). For students with short-term change, relatively more experienced short-term improvement (n = 34) versus short-term decrease (n = 23, returned to baseline performance). Approximately similar numbers of students experienced a long-term improvement from exam 2 onwards (n = 31) versus a later improvement on exams 3/4 (n = 26). Finally, for students with an overall decrease in exam grade, there were approximately similar numbers of students experiencing an immediate decrease (n = 24) versus a later decrease (n = 22).

### Changes within students’ use of study strategies

Students who were in Groups 1, 2, and 3 on exam 1 (aggregate, n = 258) reported significantly increasing their *use of course materials* from exam 1 to exam 2 (SRL1 median = 13.00, SRL2 median = 14.00; V = 4803.5, *p* < 0.0001), but not of *housekeeping strategies* (medians = 11.00; V = 8453, *p* = 0.06252) or *metacognitive strategies* (medians = 15.00; V = 11268, *p* = 0.8263). The increase in students’ use of course materials appears mostly due to the increased use of *using practice exams* specifically—for SRL1, 79.8% of these students (n = 206) reported higher use; for SRL2, 90.3% of them (n = 233) reported higher use. There was also a notable increase in *reviewing the textbook and screencasts* (79.8% for SRL1 (n = 206); 85.3% for SRL2 (n = 220)).

**Fig 4:**
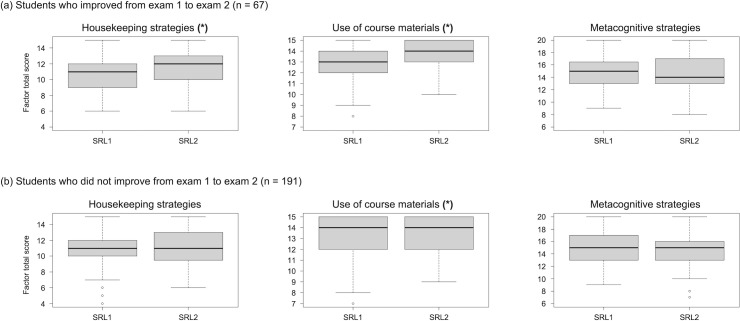
Students who improved from exam 1 to exam 2 (n = 67, panel “a”) significantly increased their use of housekeeping strategies and course materials from the SRL1 to SRL2 survey; students who did not improve (n = 191, panel “b”) significantly increased their use of course materials only. Median scores for self-reported use of *housekeeping strategies*, *course materials*, *and metacognitive strategies* on surveys SRL1 and SRL2, for the subsets of students from Groups 1, 2, and 3 who (a) improved from exam 1 to exam 2 (n = 67) versus (b) did not improve from exam 1 to exam 2 (n = 191). *Housekeeping strategies* and *use of course materials* each contain three strategies (maximum possible score of 15) whereas *metacognitive strategies* contain four (maximum possible score of 20). An asterisk in parentheses indicates a significant difference in strategy use for a given subset of students (Wilcoxon signed-ranked tests).

On the other hand, students who improved from exam 1 to exam 2 (n = 67) significantly increased both their use of *housekeeping strategies* (SRL1 median = 11.00, SRL2 median = 12.00; V = 306, *p* = 0.003464) and *use of course materials* (SRL1 median = 13.00, SRL2 median = 14.00; V = 233, *p* < 0.0001; Fig 4A). These significant increases are mostly due to students who improved from Groups 1 and 2 (n = 38: 20 from Group 1, and 18 from Group 2), who increased their median scores for these factors by 1 to 1.5 points (SRL1 *housekeeping* median = 11.00, SRL2 *housekeeping* median = 12.00, V = 61, *p* = 0.001959; SRL1 *use of course materials* median = 13.00, SRL2 *use of course materials* median = 14.5, V = 58.5, *p* < 0.001). These students also had more room for increasing use of these approaches compared to students who improved from Group 3 (n = 29), who did not significantly change in their median scores for any strategy factors (*housekeeping strategies*: *V* = 92, *p* = 0.4176; *use of course materials*: *V* = 61, *p* = 0.09757; *metacognitive strategies*: *V* = 164, *p* = 0.4297; see Fig 2). There was a median-point decrease in use of *metacognitive strategies* for students from Groups 1/2/3 who improved, yet this change was not significant (SRL1 median = 15.00, SRL2 median = 14.00; V = 799, *p* = 0.807; Fig 4A).

For comparison, students who did not improve from exam 1 to exam 2 (n = 191: 28 from Group 1, 63 from Group 2, and 100 from Group 3) significantly increased their reported *use of course materials* only (SRL1 median = 14.00, SRL2 median = 14.00; V = 2867, *p* = 0.001028; Fig 4B). While the medians on both surveys are the same, a decrease in the range of scores on SRL2 seems to drive the significant difference, in which the minimum score was 7.00 on SRL1 and 9.00 on SRL2 (same maximum score of 15.00 on both surveys)—that is, fewer students had lower scores on SRL2. Median scores for *housekeeping strategies* (11.00, V = 5489, *p* = 0.6403) and *metacognitive strategies* (15.00, V = 6115.5, *p* = 0.8992) were the same, with comparable score ranges, on both surveys (Fig 4B).

## Discussion

Biology education research is increasingly focusing on students’ use of learning strategies because strategy use is central to self-regulated learning, and can be coached through interventions that improve outcomes in STEM. Several studies contribute to a growing understanding of how specific study strategies relate to achievement [51, 53, 55, 91, 92], whether course-level interventions can promote the use of effective learning strategies and lead to better outcomes [52, 54, 77, 78, 93], and whether students change their strategies and under what circumstances they do so [60, 74, 94–98].

In this work, we investigated what learning strategies introductory biology students frequently reported using concurrently, and whether these strategies grouped into meaningful patterns. We also tracked individual students’ self-reported strategy use and exam performance over the course of one semester to determine whether students’ patterns of strategy use changed, and whether strategy changes corresponded to changes in exam performance. Our work adds to the growing body of literature in this area by showing that introductory biology students in our course (a) concurrently used multiple, related strategies to study, (b) partly shifted strategy use over the course of the semester, and in some cases, (c) achieved improved exam performance that was, in part, associated with their study strategies.

### Exam 1 score predicts successive exam scores

In this work, we used exam scores as a measure of achievement. We found that, in our course, students’ exam 1 percent score strongly predicted the average percent score of their successive exams (Fig 1), and that, for most students, percent exam scores did not deviate significantly over time despite some short-term oscillations (Fig 3). This finding is consistent with previous work showing an association between early exam performance and final course grades in undergraduate introductory biology [18] and chemistry [13]. While other studies used final course grades, we chose to analyze students’ scores on unit exams because, in our setting, final course grades included other potentially confounding components (e.g., homework, classwork, laboratory grade). We are aware that exam scores lack information about the specific concepts and skills assessed, and therefore do not accurately represent students’ learning; performance and learning are, at best, related [99]. However, we used exam scores as a metric because they are meaningful indicators of course standing to students, and they are consequential since they account for a substantial portion of the final course grade.

The association between exam 1 percent score and average percent score on exams 2/3/4 persisted in multiple iterations of the course (four semesters in total, different student cohorts; S2 Table and S3 Fig), confirming that, in our setting, exam 1 grades are strong predictors of overall course achievement. But this predictive relationship–as with any predictive model–does not hold true for *every* student. Our data clearly show there is wide variation in students’ exam 1 scores (Fig 1) and that exam 1 scores fail to account for roughly a quarter of the variance in later exam scores. The latter observation is particularly important because it tells us that, when there is room for improvement, some learners “break the mold”: they deviate from the achievement trajectory predicted by the first exam and improve upon exam 1 by at least one standard deviation (Fig 3). In this study, we examined whether students’ behaviors—their self-reported use of specific study strategies—could explain variation in exam 1 scores and improvement in exam scores over time.

### Students’ self-reported strategies clustered into three groups

Previous studies on the relationship between learning strategies and achievement in college STEM courses identified associations between individual study strategies and academic performance [51–53, 55, 91]. Specific strategies used by learners have been associated in the literature with deep or surface approaches to learning [59, 63, 64, 100]. Hattie and Donoghue’s model [58, 59] explicitly linked suites of study strategies to phases of learning that reflect different levels of cognitive and metacognitive engagement (surface, deep, transfer). According to this idea, we anticipated that our students may report using multiple strategies concurrently, and we asked which strategies, if any, would be more often reported together. The EFA we performed yielded three groups of frequently co-reported strategies (three factors; Table 2): (1) organizing and transforming, goal-setting and planning, and rehearsing and memorizing; (2) reviewing notes, using practice exams, and reviewing the textbook/screencasts; and (3) self-evaluation, reviewing graded work, monitoring understanding, and seeking instructor assistance. We named the factors based on commonalities among the items comprising each factor. We thought of organizing and transforming, goal-setting and planning, and rehearsing and memorizing (clustered in factor 1) as *housekeeping strategies*: “baseline” activities most of our students reported performing when studying. We referred to the second factor as *use of course materials* because all three strategies in this group relate to students’ direct engagement with learning materials, including course-specific resources such as instructor-made screencasts and past years’ exams. Finally, we named the third factor *metacognitive strategies* because it included strategies reflecting efforts to assess one’s understanding, evaluate progress, and learn from mistakes (self-evaluation, reviewing graded work, and monitoring understanding). Interestingly, a fourth strategy, *seeking instructor assistance*, shared variance with these three more distinctly metacognitive strategies, and was therefore included in this factor. One possible interpretation for this finding is that students who often visit instructor office hours may be generally reflective and monitor their understanding; therefore, they are aware of what they do and do not understand, and they generate questions to bring to the instructor. Contrarily, students who could benefit from instructor assistance but do not frequently review their graded work or monitor their understanding may not have specific questions to ask, and therefore do not use office hours frequently (Fig 2 and S3 Table).

The three groups of strategies (factors) emerging from the EFA aligned with the types of strategies Hattie and Donoghue had associated with surface and deep learning phases in their model [77]: *housekeeping strategies* and *use of course materials* mapped onto the surface learning phase (in which learners build foundational knowledge), and *metacognitive strategies* mapped onto the deep learning phase (in which learners refine and evaluate knowledge, problem-solve, and seek others’ help; S3 Fig). One possible interpretation of this correspondence is that what our students do to prepare for exams is related to, or even indicative of, their cognitive and metacognitive engagement with the material. Students who primarily or exclusively adopt surface-level study strategies *may* be mostly working toward building their knowledge base, acquiring key concepts and vocabulary, and possibly also developing their study habits. Conversely, we may hypothesize that students who adopt metacognitive strategies are engaging with the material on a deeper level, possibly because they came to the course with some background knowledge that enables them to learn (or review) facts and concepts more quickly, leaving room for engaging thoughtfully with higher-order cognitive tasks. As we know, however, strategy choice is guided by multiple variables, including prior experience (what strategies students have used before and are already familiar with), motivation to learn, and students’ understanding of the criteria for success in a course. It is highly likely, in our view, that students’ familiarity with course content, prior experience with study strategies, and understanding of assessment demands contributed to influence the strategy groupings that emerged from the EFA. Essentially, our surveys and EFA findings tell us what patterns of strategies our students use, but do not necessarily tell us why. Further research, possibly through targeted follow-up survey questions or structured interviews, would be necessary to get to the heart of why some students use specific groups of strategies: what drives their choice of how to study, and to what extent multiple factors contribute to shaping a student’s approach to learning and studying.

### Use of course materials and metacognitive strategies associated with higher Exam 1 scores

Students who achieved better outcomes on exam 1 reported more frequent *use of course materials* and use of *metacognitive strategies* (Fig 2). The strategies comprising these factors were also significantly associated with exam 1 scores when analyzed individually, both in this study (S3 Table) and in our previous work [53]. These findings, viewed through the lens of Hattie and Donoghue’s model of learning [59], would suggest that students who earned high exam 1 scores prepared for the exam by concurrently using strategies aimed at *consolidating surface learning* (reviewing course records and taking practice exams), and strategies associated with *deep learning* (such as critically reviewing graded work and seeking instructor help). From our perspective, these findings are consistent with what we would expect in a first-semester college introductory biology course in which students need to learn key facts and ideas but are also tasked with analyzing problems and applying concepts. Students in the course were encouraged to acquire facts and vocabulary for each topic through a flipped instructional design (see Methods), and they practiced applying their understanding through in-class activities and homework problems that mirrored the types of questions they would encounter on exams. Therefore, it was not surprising to us that a combination of surface and deep approaches, aimed at building a knowledge base and using that knowledge, would be associated with high exam performance.

Cognizant that clear learning objectives and practice assessments signal to students what they are expected to know and do and, consequently, can influence how students study [60, 66, 67], course instructors included learning objectives in all lessons and shared previous years’ exam questions. We hypothesize that *using practice exams* (one of the strategies included in *use of course materials*) was particularly critical in preparing for exam 1 since it informed students of what kinds of questions to expect on the test. *Seeking instructor assistance*, a strategy that clustered with *metacognitive strategies* in the EFA, was also significantly associated with higher exam 1 scores, despite being the least-frequently reported strategy for exam 1. This finding is consistent with our own prior work and other studies, showing that higher-achieving students tend to seek social assistance, and ask the instructor for help more often [47, 53, 101].

In our prior study, we had combined *keeping records and monitoring* into a single survey item, and this combined strategy was associated with higher exam grades. To achieve better resolution, in this study, we replaced that item with two distinct strategies: *keeping records* and *monitoring understanding*. We found that these two strategies were reported by our students with different frequencies, and only *monitoring understanding* was statistically associated with higher exam grades. This outcome underscores that the two strategies are in fact distinct and should not be merged into a single survey item. The fact that *monitoring understanding* (a metacognitive strategy that can promote deep learning [59]) is associated with high achievement in our course context, while *keeping records* (a surface-learning strategy) is not, merits some interpretation. *Keeping records* is unlikely to be ineffective or unimportant; rather, it may be a foundational (*housekeeping*) study strategy that is equally used by most students in our course, and therefore does not discriminate among higher and lower exam scores. *Monitoring understanding*, on the other hand, is less common and may reflect a deeper approach to learning that leads to higher achievement in our course context.

### Increasing housekeeping strategies and use of course materials associated with improved exam scores

Students in our course reported an overall increased *use of course materials* (specifically, previous years’ exams, and the textbook or instructor screencasts) when studying for exam 2, compared to exam 1. Students whose scores improved from exam 1 to exam 2, however, also specifically reported increasing their use of *housekeeping strategies*, such as planning their studying, and rehearsing and memorizing (Fig 4B).

Changes in patterns of strategy use are expected: as students engage with a course and better understand its demands, they may find it necessary to change their approaches to meet these demands and achieve their goals [60, 98]. However, to our knowledge, specific changes in terms of strategy use had not yet been empirically associated with improvement on course exams in introductory biology. This study, therefore, begins to shed light on what students may do that enables them to “break the mold” and become more proficient on biology exams.

It may feel counterintuitive that exam grade improvement, in our course, was associated with increased use of *housekeeping strategies* and *use of course materials*, but not of *metacognitive strategies*–especially considering that *metacognitive strategies* were associated with higher scores on exam 1. A possible reason is that learners who earned low scores on exam 1, and wanted to improve, started by building up their baseline study habits. They may have focused on intensifying their efforts through *housekeeping strategies* aimed at acquiring and consolidating knowledge. Because our course exams included a variable but significant proportion of questions focused on lower-order cognitive skills (knowledge and understanding), increased acquisition and consolidation of surface knowledge would be sufficient to yield some grade improvement. *Metacognitive strategies*, on the other hand, not only involve self-regulation, but also require sufficient background knowledge for the learner to be able to identify knowledge gaps, evaluate their own work and that of others, grasp detail and nuance in concepts, and seek help if necessary [59]. It is possible that students seeking to improve would begin building toward better outcomes by increasing effort with practices that are (a) straightforward and familiar and (b) aimed at building a foundation of knowledge which is necessary for further, deeper reasoning. More research is necessary to test this hypothesis: future work, for instance, could identify the cognitive level of exam items (differentiating higher-order and lower-order cognitive skills) and relate student performance on different-level items to students’ self-reported surface versus deep learning strategies.

### Limitations and future directions

A potential limitation of our analytical approach for this study is that, rather than using raw exam scores or continuous z-scores, we chose to create z-score bins (categories). Our intent with transforming raw scores to z-scores was to (a) control for differences in content and difficulty across exams, and (b) make a clear, standardized definition of improvement that accounted for students’ performance on exam 1 as a baseline. The purpose of binning was to create categories that provided an adequate approximation for letter grades; the z-score groupings we used were closely aligned with the letter grades corresponding to exam score ranges (e.g., 90–100% = A, 80–89% = B etc.). We recognize that binning continuous data into categories naturally results in some loss of statistical information. We considered the trade-off between the potential benefits of obtaining fine-grained results using continuous data, and the potential risk of oversimplifying the findings by making the data categorical. While a more fine-grained analysis with continuous data would have been feasible, we were concerned about potentially identifying changes in exam scores that may be statistically significant, yet not bear practical significance in the classroom. For instance, very small changes in exam scores (2–5% change) may not be enough to result in a change in letter grades. Also, using raw percent scores, we could identify a 20% improvement on exam grades for two different students, with completely different implications in each case. On a traditional grading scale, a change from 30% to 50% (from F to F) does not carry the same meaning as a change from 50% to 70% (from F to C). This approach helped us to simplify the data and focus on changes in exam scores that would be meaningful to students in the course. From a reproducibility perspective, using z-score groupings also facilitated comparison to our previous work, which had identified an association between frequency of strategy use and exam performance as self-reported categorical letter grades [53].

Another methodological limitation was using surveys asking students to self-report how frequently they used various study strategies. Our data inform us of how frequently students reported using certain strategies, but do not provide any indication of whether students implemented strategies effectively and whether any choices and changes in strategy use were deliberate and planned [98, 102]. Knowing how and when to use a strategy (procedural and conditional knowledge of strategies, respectively) reflects metacognitive regulation of strategy use, a skill that may develop at different times in different students [74, 98]. Learners whose metacognition is still emerging, for example, may struggle to carry out effectively appropriate strategies [74]; as a result, students may report increased use of theoretically beneficial strategies, yet their outcomes may not improve (Fig 4A). Even a seemingly straightforward strategy such as “reviewing the textbook” can be implemented in different ways: rereading superficially is not the same as intentionally returning to select text sections with the intent of filling specific gaps in understanding [103]. Further research with open-ended survey questions and semi-structured interviews is needed to understand how and why students use particular strategies, and to further clarify the relationship between *strategy implementation* and achievement.

With our present data, we can only make inferences about students’ reported strategy use in relation to characteristics of our course context [66]. Although choice and effectiveness of study strategies are theoretically expected to vary depending on context [59], more evidence is necessary to identify how learners’ individual preferences and experiences and course contextual cues interact to influence students’ choice and use of strategies. Future work should use surveys and possibly interviews to document how student learning strategies may vary across course settings (e.g., in different STEM disciplines, in lecture vs. laboratory, or in lower- and upper-division courses), in relation to course features (such as assessment demands and success criteria).

### Implications for education

The findings in this study, together with those of others [52, 54, 77, 78, 92, 93], are encouraging because they suggest that students can exert some control over their outcomes by changing their study approaches. However, student behavior is only one element in the complex system of learning, which is influenced and moderated by other variables (learners’ characteristics and the learning environment). Any efforts aimed at fostering students’ adoption of appropriate study strategies need to take into account (a) *learners’ individual differences*: not all students come to our courses already knowing how to identify the best study strategies for a given context and how to use them effectively, and (b) *the environment*: without supportive course policies and structures, students may invest effort in trying to change their study strategies with little return in terms of achievement.

As a result of engaging with this research in our own classroom, we have developed greater awareness of areas of course design that we can influence as instructors, to empower learners toward developing effective study strategies and making meaningful, lasting improvement:

*(1) Define and clearly communicate expectations and criteria for success*. It is critical that we make course expectations and criteria for success clear and explicit, so students are aware of what knowledge and skills are assessed in the course and have the opportunity to plan their work accordingly [59]. We believe this clarity begins with the syllabus outlining learning outcomes, grading scale, and relevant policies. In daily practice, instructors can support students’ work toward the intended learning outcomes by (a) communicating the learning objectives of each lesson [67], (b) designing instruction and assessments that are closely aligned to the learning objectives [104], and (c) providing meaningful practice opportunities (e.g., practice exams, formative homework and class activities) followed by useful, timely feedback [105].

*(2) Identify course-appropriate study strategies and explicitly promote their use*. Hattie and Donoghue, among others, point out that, because context is critically important in mediating efficacy of strategies, study skills should not be taught in isolation, but embedded within disciplinary courses [59]. Explicit discussion and guidance on effective study strategies can encourage students to adopt them more frequently, resulting in improved outcomes in undergraduate biology courses [52, 77, 78, 93]. While instructors have some knowledge of effective strategies, their knowledge has limitations [71], and it is therefore critical to develop an evidence-based understanding of which strategies are effective in a given context. In our course, conducting post-exam surveys provided an invaluable source of information about what strategies our students used when studying for exams and what strategies seemed most beneficial in terms of exam grades [53]. We do not presume that every instructor would need to conduct a systematic investigation in their courses; however, we feel that instructors would gain a tremendous amount of information by surveying their students about what they do when they study and what works in their course. Surveys can be created ad-hoc or adapted from published studies (e.g., the SRL survey used in this work, and others [55, 94, 95]). Alternatively, instructors may use class discussions or focus groups to gather evidence. Once instructors have identified which strategies are best suited for achieving their course outcomes, they can deliberately coach students on using these specific strategies through targeted discussions during lecture or laboratory sessions [52, 78], written reflection activities [77, 78], and co-curricular workshops tailored to the specific course [93].

*(3) When possible*, *supplement generalized study strategy advice with personalized advice*. There is some evidence that not all students are ready to adopt and implement effectively strategies associated with deeper learning [60]. It becomes critical, therefore, that instructors engage with struggling students individually to tailor advice more specifically for their needs. Finding out what a student does to study, what their background knowledge is, and what strategies they are already familiar with may be extremely helpful in finding a good starting point for them, if suggesting change. Personalized guidance, for instance, may help a student prioritize learning tasks to first build baseline knowledge and then use some metacognitive approaches to evaluate their progress, fill gaps, and resolve misconceptions, thus laying the groundwork for higher-order cognitive tasks. We are aware that this type of conversation may not be feasible in very large course settings; yet, we have students who come to talk to us about wanting to improve, and those occasions open the door to fruitful dialogue about how they study and what they may be doing differently going forward.

*(4) Create room for improvement by transforming grading practices*. Although teaching students to use effective study strategies can help them grow as learners and study more effectively, the design of learning environments can still pose significant barriers to achievement and improvement. Our finding that exam 1 scores closely predicted the scores on subsequent exams (Figs 1 and S2) was very eye-opening and led us to consider the impact of our grading practices on student outcomes. In our course, we used a traditional grading system (see Methods) in which each unit exam accounted for a predetermined proportion of the final course grade. Given the range of improvement we observed on exams, however, it follows that students who performed poorly on exam 1 would very likely be mathematically excluded from earning a high grade in the course, even if they improved on subsequent exams. Our evidence lends further support to the idea that traditional grading systems that designate most of the scale (0–60%) as a failing grade and assign fixed weights to exams greatly limit students’ chances of significantly improving their course grades after a rough start. Faced with a shrinking ceiling to how much they can improve their outcomes, struggling students may not invest in changing their strategies at all. Traditional grading systems, therefore, undermine student effort and persistence and perpetuate inequities in education [106]. Confronted with our own evidence and encouraged by the growing conversation about alternative grading practices, we have been working to transform our course by adopting an alternative grading approach (standards-based grading) that provides opportunities for students to learn from their mistakes, engage more productively and proactively in their learning, and reap the benefits of their work, as improvement is reflected in their grades [106–108]. As more insights on equitable grading practices in college biology begin to emerge [109, 110], we believe that reshaping the norms surrounding grading may be one of the key elements in a learning environment that enables learners to grow and succeed–in other words, to “break the mold.”

## Supporting information

S1 Table Students’ self-reported demographic background information and school status (n = 376). (PDF)

S2 Table Frequency with which students who earned different scores on Exam 1 (based on z-score groupings) reported higher use (Often/Very often) of each of the 17 SRL study strategies on the SRL1 survey. Strategies are listed from most to least used, based on the overall sample’s responses. The χ² value from the contingency analysis is reported for each strategy (*df* = 3; adjusted α = 0.002941). Strategies having a significant association with exam score are highlighted, and boldfaced χ² values indicate statistical significance (*p* < 0.0001 for *reviewing graded work*, *self-evaluation*, *using practice exams*, *monitoring understanding*; *p* < 0.001 for *reviewing textbook/screencasts*, *seeking instructor assistance*. The *p*-values for the remaining strategies range from 0.0222 (*goal-setting and planning*) to 0.834 (*self-consequating*).
(PDF)

S3 Table Post-hoc Mann-Whitney U test results for pairwise comparisons, strategy factor scores by Exam 1 z-score groups. Bonferroni correction applied for six pairwise tests per strategy factor; adjusted α = 0.008333.
(PDF)

S1 File Self-regulated learning strategies survey, administered after Exam 1 (SRL1) and Exam 2 (SRL2). (PDF)

S2 File Exam and SRL survey dataset for the primary course cohort. (CSV)

S3 File Means, standard error, and linear regression equation and statistical results for Exam 1 and the average of Exams 2/3/4 for three additional, consecutive course cohorts. (PDF)

S4 File Exam data for three additional, consecutive course cohorts. (CSV)

S1 Fig Schematic for the z-score groupings, which were used to distinguish improvement versus non-improvement on exams over time. (PDF)

S2 Fig Exam 1 strongly and significantly predicts subsequent exam performance (the average percent score of Exams 2, 3, and 4), for three additional, consecutive cohorts of the introductory biology course of study. (A) Linear regression for Year 1 cohort, (B) linear regression for Year 2 cohort, and (C) linear regression for Year 3 cohort. The equation and R^2^ value for each cohort are also provided, as well as the 95% confidence interval for the regression line.
(PDF)

S3 Fig Strategy factors mapped onto the model of learning proposed by Hattie and Donoghue (2016). The outlined boxes represent the learning phases in Hattie and Donoghue’s model. Light blue-shaded boxes represents the factors emerging from our EFA, each listing the study strategies that loaded onto each factor.
(PDF)

## References

[1] Chen X. STEM Attrition: College Students’ Paths into and out of STEM Fields. Statistical Analysis Report. NCES 2014–001. National Center for Education Statistics. 2013.

[2] Thiry H, Weston TJ, Harper RP, Holland DG, Koch AK, Drake BM, et al. Talking about Leaving Revisited: Persistence Relocation, and Loss in Undergraduate STEM Education. Hunter A-B, Seymour E, editors. Switzerland: Springer; 2019. 528 p.

[3] Daempfle PA. An Analysis of the High Attrition Rates among First Year College Science, Math, and Engineering Majors. Journal of College Student Retention: Research, Theory & Practice. 2003;5(1):37–52.

[4] Gasiewski JA, Eagan MK, Garcia GA, Hurtado S, Chang MJ. From Gatekeeping to Engagement: A Multicontextual, Mixed Method Study of Student Academic Engagement in Introductory STEM Courses. Research in Higher Education. 2012;53(2):229–61. doi: 10.1007/s11162-011-9247-y 23503751 PMC3596160

[5] Schneider M, Preckel F. Variables associated with achievement in higher education: A systematic review of meta-analyses. Psychological bulletin. 2017;143(6):565. doi: 10.1037/bul0000098 28333495

[6] Richardson M, Abraham C, Bond R. Psychological correlates of university students’ academic performance: A systematic review and meta-analysis. Psychological Bulletin. 2012;138(2):353–87. doi: 10.1037/a0026838 22352812

[7] Bandura A. Social foundations of thought and action: A social cognitive theory. Social foundations of thought and action: A social cognitive theory: Prentice-Hall, Inc; 1986. p. xiii, 617-xiii,.

[8] Schunk DH. Learning theories: An educational perspective. 6th ed. Boston MA: Pearson; 2012.

[9] Strayhorn TL. Modeling the Determinants of College Readiness for Historically Underrepresented Students at 4-Year Colleges and Universities:A National Investigation. American Behavioral Scientist. 2014;58(8):972–93.

[10] Westrick PA, Le H, Robbins SB, Radunzel JMR, Schmidt FL. College Performance and Retention: A Meta-Analysis of the Predictive Validities of ACT® Scores, High School Grades, and SES. Educational Assessment. 2015;20(1):23–45.

[11] Westrick PA, Schmidt FL, Le H, Robbins SB, Radunzel JMR. The Road to Retention Passes through First Year Academic Performance: A Meta-Analytic Path Analysis of Academic Performance and Persistence. Educational Assessment. 2021;26(1):35–51.

[12] Harackiewicz JM, Canning EA, Tibbetts Y, Priniski SJ, Hyde JS. Closing achievement gaps with a utility-value intervention: Disentangling race and social class. Journal of Personality and Social Psychology. 2016;111(5):745–65. doi: 10.1037/pspp0000075 26524001 PMC4853302

[13] Thompson ME. Grade Expectations: The Role of First-Year Grades in Predicting the Pursuit of STEM Majors for First- and Continuing-Generation Students. The Journal of Higher Education. 2021;92(6):961–85.

[14] Ballen CJ, Salehi S, Cotner S. Exams disadvantage women in introductory biology. PLOS ONE. 2017;12(10):e0186419. doi: 10.1371/journal.pone.0186419 29049334 PMC5648180

[15] Ballen CJ, Wieman C, Salehi S, Searle JB, Zamudio KR. Enhancing Diversity in Undergraduate Science: Self-Efficacy Drives Performance Gains with Active Learning. CBE—Life Sciences Education. 2017;16(4):ar56. doi: 10.1187/cbe.16-12-0344 29054921 PMC5749958

[16] Harackiewicz JM, Canning EA, Tibbetts Y, Giffen CJ, Blair SS, Rouse DI, et al. Closing the social class achievement gap for first-generation students in undergraduate biology. Journal of Educational Psychology. 2014;106(2):375–89. doi: 10.1037/a0034679 25049437 PMC4103196

[17] Dai T, Cromley JG. Changes in implicit theories of ability in biology and dropout from STEM majors: A latent growth curve approach. Contemporary Educational Psychology. 2014;39(3):233–47.

[18] Jensen PA, Barron JN. Midterm and First-Exam Grades Predict Final Grades in Biology Courses. Journal of College Science Teaching. 2014;44(2):82–9.

[19] Mills P, Sweeney W, Bonner SM. Using the First Exam for Student Placement in Beginning Chemistry Courses. Journal of Chemical Education. 2009;86(6):738.

[20] Lee UJ, Sbeglia GC, Ha M, Finch SJ, Nehm RH. Clicker Score Trajectories and Concept Inventory Scores as Predictors for Early Warning Systems for Large STEM Classes. Journal of Science Education and Technology. 2015;24(6):848–60.

[21] Easterbrook MJ, Hadden IR. Tackling Educational Inequalities with Social Psychology: Identities, Contexts, and Interventions. Social Issues and Policy Review. 2021;15(1):180–236.

[22] Freeman S, Eddy SL, McDonough M, Smith MK, Okoroafor N, Jordt H, et al. Active learning increases student performance in science, engineering, and mathematics. Proceedings of the National Academy of Sciences. 2014;111(23):8410–5. doi: 10.1073/pnas.1319030111 24821756 PMC4060654

[23] Kara E, Tonin M, Vlassopoulos M. Class size effects in higher education: Differences across STEM and non-STEM fields. Economics of Education Review. 2021;82:102104.

[24] Scott AN, McNair DE, Lucas JC, Land KM. From Gatekeeper to Gateway: Improving Student Success in an Introductory Biology Course. Journal of College Science Teaching. 2017;46(4):93–9.

[25] Solanki S, McPartlan P, Xu D, Sato BK. Success with EASE: Who benefits from a STEM learning community? PLOS ONE. 2019;14(3):e0213827. doi: 10.1371/journal.pone.0213827 30901339 PMC6430422

[26] Brewer CA, Smith D. Vision and change in undergraduate biology education: a call to action. American Association for the Advancement of Science, Washington, DC. 2011;81.

[27] Ambrose SA, Bridges MW, DiPietro M, Lovett MC, Norman MK. How learning works: Seven research-based principles for smart teaching: John Wiley & Sons; 2010.

[28] Freeman S, Haak D, Wenderoth MP. Increased Course Structure Improves Performance in Introductory Biology. CBE—Life Sciences Education. 2011;10(2):175–86. doi: 10.1187/cbe.10-08-0105 21633066 PMC3105924

[29] Eddy SL, Hogan KA. Getting Under the Hood: How and for Whom Does Increasing Course Structure Work? CBE—Life Sciences Education. 2014;13(3):453–68. doi: 10.1187/cbe.14-03-0050 25185229 PMC4152207

[30] Haak D, HilleRisLambers J, Pitre E, Freeman S. Increased Structure and Active Learning Reduce the Achievement Gap in Introductory Biology. Science. 2011;332(6034):1213–6. doi: 10.1126/science.1204820 21636776

[31] Theobald EJ, Hill MJ, Tran E, Agrawal S, Arroyo EN, Behling S, et al. Active learning narrows achievement gaps for underrepresented students in undergraduate science, technology, engineering, and math. Proceedings of the National Academy of Sciences. 2020;117(12):6476–83. doi: 10.1073/pnas.1916903117 32152114 PMC7104254

[32] Dewsbury BM, Swanson HJ, Moseman-Valtierra S, Caulkins J. Inclusive and active pedagogies reduce academic outcome gaps and improve long-term performance. PLOS ONE. 2022;17(6):e0268620. doi: 10.1371/journal.pone.0268620 35704639 PMC9200326

[33] Bailey EG, Jensen J, Nelson J, Wiberg HK, Bell JD. Weekly Formative Exams and Creative Grading Enhance Student Learning in an Introductory Biology Course. CBE—Life Sciences Education. 2017;16(1):ar2. doi: 10.1187/cbe.16-02-0104 28130269 PMC5332045

[34] Bliuc A-M, Ellis RA, Goodyear P, Hendres DM. Understanding student learning in context: relationships between university students’ social identity, approaches to learning, and academic performance. European Journal of Psychology of Education. 2011;26(3):417–33.

[35] González A, Paoloni P-V. Perceived autonomy-support, expectancy, value, metacognitive strategies and performance in chemistry: a structural equation model in undergraduates. Chemistry Education Research and Practice. 2015;16(3):640–53.

[36] Partin ML, Haney JJ. The CLEM model: Path analysis of the mediating effects of attitudes and motivational beliefs on the relationship between perceived learning environment and course performance in an undergraduate non-major biology course. Learning Environments Research. 2012;15(1):103–23.

[37] Robbins SB, Oh I-S, Le H, Button C. Intervention effects on college performance and retention as mediated by motivational, emotional, and social control factors: Integrated meta-analytic path analyses. Journal of Applied Psychology. 2009;94(5):1163–84. doi: 10.1037/a0015738 19702363

[38] Zander L, Brouwer J, Jansen E, Crayen C, Hannover B. Academic self-efficacy, growth mindsets, and university students’ integration in academic and social support networks. Learning and Individual Differences. 2018;62:98–107.

[39] Bandura A. Social cognitive theory of self-regulation. Organizational Behavior and Human Decision Processes. 1991;50(2):248–87.

[40] Zimmerman BJ. A social cognitive view of self-regulated academic learning. Journal of Educational Psychology. 1989;81(3):329–39.

[41] Zimmerman BJ. Becoming a self-regulated learner: An overview. Theory into practice. 2002;41(2):64–70.

[42] Zimmerman BJ. Self-Regulated Learning and Academic Achievement: An Overview. Educational Psychologist. 1990;25(1):3–17.

[43] Panadero E. A review of self-regulated learning: Six models and four directions for research. Frontiers in psychology. 2017:422. doi: 10.3389/fpsyg.2017.00422 28503157 PMC5408091

[44] Nilson LB. Creating self-regulated learners: Strategies to strengthen students’ self-awareness and learning skills: Stylus Publishing, LLC; 2013.

[45] Schunk DH, Zimmerman BJ. Self‐Regulation and Learning. Handbook of Psychology. 2003:59–78.

[46] Dignath C, Büttner G. Components of fostering self-regulated learning among students. A meta-analysis on intervention studies at primary and secondary school level. Metacognition and learning. 2008;3:231–64.

[47] Zimmerman BJ, Pons MM. Development of a Structured Interview for Assessing Student Use of Self-Regulated Learning Strategies. American Educational Research Journal. 1986;23(4):614–28.

[48] Hartwig MK, Dunlosky J. Study strategies of college students: Are self-testing and scheduling related to achievement? Psychonomic Bulletin & Review. 2012;19(1):126–34. doi: 10.3758/s13423-011-0181-y 22083626

[49] Dunlosky J, Rawson KA, Marsh EJ, Nathan MJ, Willingham DT. Improving students’ learning with effective learning techniques: Promising directions from cognitive and educational psychology. Psychological Science in the Public interest. 2013;14(1):4–58. doi: 10.1177/1529100612453266 26173288

[50] Credé M, Kuncel NR. Study Habits, Skills, and Attitudes: The Third Pillar Supporting Collegiate Academic Performance. Perspectives on Psychological Science. 2008;3(6):425–53. doi: 10.1111/j.1745-6924.2008.00089.x 26158971

[51] Kritzinger A, Lemmens J-C, Potgieter M. Learning Strategies for First-Year Biology: Toward Moving the “Murky Middle”. CBE—Life Sciences Education. 2018;17(3):ar42. doi: 10.1187/cbe.17-10-0211 30142052 PMC6234818

[52] Rodriguez F, Rivas MJ, Matsumura LH, Warschauer M, Sato BK. How do students study in STEM courses? Findings from a light-touch intervention and its relevance for underrepresented students. PLOS ONE. 2018;13(7):e0200767. doi: 10.1371/journal.pone.0200767 30063744 PMC6067695

[53] Sebesta AJ, Bray Speth E. How Should I Study for the Exam? Self-Regulated Learning Strategies and Achievement in Introductory Biology. CBE—Life Sciences Education. 2017;16(2):ar30. doi: 10.1187/cbe.16-09-0269 28495934 PMC5459248

[54] Vemu S, Denaro K, Sato BK, Fisher MR, Williams AE. Moving the Needle: Evidence of an Effective Study Strategy Intervention in a Community College Biology Course. CBE—Life Sciences Education. 2022;21(2):ar24. 35544204 10.1187/cbe.21-08-0216 PMC9508909

[55] Walck-Shannon EM, Rowell SF, Frey RF. To What Extent Do Study Habits Relate to Performance? CBE—Life Sciences Education. 2021;20(1):ar6. doi: 10.1187/cbe.20-05-0091 33444109 PMC8108503

[56] Greene JA, Azevedo R. A theoretical review of Winne and Hadwin’s model of self-regulated learning: New perspectives and directions. Review of educational research. 2007;77(3):334–72.

[57] Winne PH. A cognitive and metacognitive analysis of self-regulated learning. Handbook of self-regulation of learning and performance. New York: Routledge; 2011. p. 29–46.

[58] Hattie JAC, Donoghue GM. A model of learning: Optimizing the effectiveness of learning strategies. Contemporary theories of learning: Routledge; 2018. p. 97–113.

[59] Hattie JAC, Donoghue GM. Learning strategies: a synthesis and conceptual model. npj Science of Learning. 2016;1(1):16013. doi: 10.1038/npjscilearn.2016.13 30792898 PMC6380372

[60] Dye KM, Stanton JD. Metacognition in Upper-Division Biology Students: Awareness Does Not Always Lead to Control. CBE—Life Sciences Education. 2017;16(2):ar31. doi: 10.1187/cbe.16-09-0286 28495935 PMC5459249

[61] Frey N, Fisher D, Hattie J. Surface, deep, and transfer? Considering the role of content literacy instructional strategies. Journal of Adolescent & Adult Literacy. 2017;60(5):567–75.

[62] van der Graaf J, Lim L, Fan Y, Kilgour J, Moore J, Gašević D, et al. The Dynamics Between Self-Regulated Learning and Learning Outcomes: an Exploratory Approach and Implications. Metacognition and Learning. 2022;17(3):745–71.

[63] Biggs JB. Student Approaches to Learning and Studying: Research Monograph. 1987.

[64] Entwistle N, Tait H. Approaches to learning, evaluations of teaching, and preferences for contrasting academic environments. Higher Education. 1990;19(2):169–94.

[65] Broekkamp H, Van Hout-Wolters BHAM. Students’ Adaptation of Study Strategies When Preparing for Classroom Tests. Educational Psychology Review. 2007;19(4):401–28.

[66] Hora MT, Oleson AK. Examining study habits in undergraduate STEM courses from a situative perspective. International Journal of STEM Education. 2017;4(1):1.

[67] Osueke B, Mekonnen B, Stanton JD. How Undergraduate Science Students Use Learning Objectives to Study. Journal of Microbiology & Biology Education. 2018; 19(2):19.2.30. doi: 10.1128/jmbe.v19i2.1510 29983848 PMC6022773

[68] Ross ME, Green SB, Salisbury-Glennon JD, Tollefson N. College Students’ Study Strategies as a Function of Testing: An Investigation into Metacognitive Self-Regulation. Innovative Higher Education. 2006;30(5):361–75.

[69] Scouller K. The influence of assessment method on students’ learning approaches: Multiple choice question examination versus assignment essay. Higher Education. 1998;35(4):453–72.

[70] Wilson K, Fowler J. Assessing the impact of learning environments on students’ approaches to learning: comparing conventional and action learning designs. Assessment & Evaluation in Higher Education. 2005;30(1):87–101.

[71] Morehead K, Rhodes MG, DeLozier S. Instructor and student knowledge of study strategies. Memory. 2016;24(2):257–71. doi: 10.1080/09658211.2014.1001992 25625188

[72] Karpicke JD, Butler AC, Roediger HL III. Metacognitive strategies in student learning: Do students practise retrieval when they study on their own? Memory. 2009;17(4):471–9. doi: 10.1080/09658210802647009 19358016

[73] Kornell N, Bjork RA. Learning Concepts and Categories:Is Spacing the “Enemy of Induction”? Psychological Science. 2008;19(6):585–92. 18578849 10.1111/j.1467-9280.2008.02127.x

[74] Stanton JD, Neider XN, Gallegos IJ, Clark NC. Differences in Metacognitive Regulation in Introductory Biology Students: When Prompts Are Not Enough. CBE—Life Sciences Education. 2015;14(2):ar15. doi: 10.1187/cbe.14-08-0135 25976651 PMC4477731

[75] Wingate U. A Framework for Transition: Supporting ‘Learning to Learn’ in Higher Education. Higher Education Quarterly. 2007;61(3):391–405.

[76] McGuire SY. Teach students how to learn: Strategies you can incorporate into any course to improve student metacognition, study skills, and motivation: Stylus Publishing, LLC; 2015.

[77] Hecht CA, Latham AG, Buskirk RE, Hansen DR, Yeager DS. Peer-Modeled Mindsets: An Approach to Customizing Life Sciences Studying Interventions. CBE—Life Sciences Education. 2022;21(4):ar82. doi: 10.1187/cbe.22-07-0143 36282273 PMC9727603

[78] Hensley L, Kulesza A, Peri J, Brady AC, Wolters CA, Sovic D, et al. Supporting Undergraduate Biology Students’ Academic Success: Comparing Two Workshop Interventions. CBE—Life Sciences Education. 2021;20(4):ar60. doi: 10.1187/cbe.21-03-0068 34605666 PMC8715789

[79] Hattie J, Biggs J, Purdie N. Effects of learning skills interventions on student learning: A meta-analysis. Review of educational research. 1996;66(2):99–136.

[80] Seery MK. Flipped learning in higher education chemistry: Emerging trends and potential directions. Chemistry Education Research and Practice. 2015;16(4):758–68.

[81] Reinagel A, Bray Speth E. Beyond the Central Dogma: Model-Based Learning of How Genes Determine Phenotypes. CBE—Life Sciences Education. 2016;15(1):ar4. doi: 10.1187/cbe.15-04-0105 26903496 PMC4803093

[82] R Core Team. R: A language and environment for statistical computing. Vienna, Austria: R Foundation for Statistical Computing; 2020.

[83] Costello AB, Osborne J. Best practices in exploratory factor analysis: Four recommendations for getting the most from your analysis. Practical Assessment, Research, and Evaluation. 2005;10(1):7.

[84] Yong AG, Pearce S. A beginner’s guide to factor analysis: Focusing on exploratory factor analysis. Tutorials in Quantitative Methods for Psychology. 2013;9(2):79–94.

[85] Zygmont C, Smith MR. Robust factor analysis in the presence of normality violations, missing data, and outliers: Empirical questions and possible solutions. TQMP. 2014;10(1):40–55.

[86] Fletcher TD. QuantPsyc: Quantitative Psychology Tools. 1.6 ed2022.

[87] Komsta L. outliers: Tests for Outliers. 0.15 ed2022.

[88] Revelle W. psych: Procedures for Personality and Psychological Research. 2.1.9 ed. Evanston Illinois, USA: Northwestern University; 2021.

[89] Knekta E, Runyon C, Eddy S. One Size Doesn’t Fit All: Using Factor Analysis to Gather Validity Evidence When Using Surveys in Your Research. CBE—Life Sciences Education. 2019;18(1):rm1. doi: 10.1187/cbe.18-04-0064 30821600 PMC6757227

[90] DiStefano C, Zhu M, Mindrila D. Understanding and using factor scores: Considerations for the applied researcher. Practical Assessment, Research, and Evaluation. 2009;14(1):20.

[91] Walck-Shannon EM, Cahill MJ, McDaniel MA, Frey RF. Participation in Voluntary Re-quizzing Is Predictive of Increased Performance on Cumulative Assessments in Introductory Biology. CBE—Life Sciences Education. 2019;18(2):ar15. doi: 10.1187/cbe.18-08-0163 31025914 PMC6755221

[92] Ainscough L, Stewart E, Colthorpe K, Zimbardi K. Learning hindrances and self-regulated learning strategies reported by undergraduate students: identifying characteristics of resilient students. Studies in Higher Education. 2018;43(12):2194–209.

[93] Hawkins W, Goddard K, Favero C. A Cocurricular Program That Encourages Specific Study Skills and Habits Improves Academic Performance and Retention of First-Year Undergraduates in Introductory Biology. CBE—Life Sciences Education. 2021;20(1):ar4. doi: 10.1187/cbe.20-06-0117 33444102 PMC8108492

[94] Andaya G, Hrabak VD, Reyes ST, Diaz RE, McDonald KK. Examining the Effectiveness of a Postexam Review Activity to Promote Self-Regulation in Introductory Biology Students. Journal of College Science Teaching. 2017;46(4):84–92.

[95] Dang NV, Chiang JC, Brown HM, McDonald KK. Curricular Activities that Promote Metacognitive Skills Impact Lower-Performing Students in an Introductory Biology Course. Journal of Microbiology & Biology Education. 2018;19(1):19.1.0. 10.1128/jmbe.v19i1.1324 PMC5969437 29904551

[96] Metzger KJ, Smith BA, Brown E, Soneral PA. SMASH: A diagnostic tool to monitor student metacognition, affect, and study habits in an undergraduate science course. Journal of College Science Teaching. 2018;47(3):88–99.

[97] Smith BA, Metzger K, Soneral P. Investigating Introductory Nonmajor Biology Students’ Self-Regulated Learning Strategies Through the Implementation of a Reflective-Routine. Journal of College Science Teaching. 2019;48(6):66–76.

[98] Stanton JD, Morris Dye K, Johnson MS. Knowledge of Learning Makes a Difference: A Comparison of Metacognition in Introductory and Senior-Level Biology Students. CBE—Life Sciences Education. 2019;18(2):ar24. doi: 10.1187/cbe.18-12-0239 31144572 PMC6755210

[99] Soderstrom NC, Bjork RA. Learning versus performance: An integrative review. Perspectives on Psychological Science. 2015;10(2):176–99. doi: 10.1177/1745691615569000 25910388

[100] Gettinger M, Seibert JK. Contributions of Study Skills to Academic Competence. School Psychology Review. 2002;31(3):350–65.

[101] Karabenick SA, Knapp JR. Relationship of academic help seeking to the use of learning strategies and other instrumental achievement behavior in college students. Journal of Educational Psychology. 1991;83(2):221–30.

[102] Blasiman RN, Dunlosky J, Rawson KA. The what, how much, and when of study strategies: comparing intended versus actual study behaviour. Memory. 2017;25(6):784–92. doi: 10.1080/09658211.2016.1221974 27561889

[103] Kuhbandner C, Emmerdinger KJ. Do students really prefer repeated rereading over testing when studying textbooks? A reexamination. Memory. 2019;27(7):952–61. doi: 10.1080/09658211.2019.1610177 31045483

[104] Wiggins GP, McTighe J. Understanding by design. Alexandria, Va.: Association for Supervision and Curriculum Development; 1998. viii, 201 p. p.

[105] Nicol DJ, Macfarlane-Dick D. Formative assessment and self-regulated learning: a model and seven principles of good feedback practice. Studies in Higher Education. 2006;31(2):199–218.

[106] Feldman J. Grading for equity: What it is, why it matters, and how it can transform schools and classrooms. Thousand Oaks, CA: Corwin; 2019. 296 p.

[107] Blum SE. Ungrading: Why rating students undermines learning (and what to do instead): UBC Press; 2020. 274 p.

[108] Townsley M, Schmid D. Alternative grading practices: An entry point for faculty in competency‐based education. The Journal of Competency‐Based Education. 2020;5(3):e01219.

[109] Cotner S, Ballen CJ. Can mixed assessment methods make biology classes more equitable? PLoS One. 2017;12(12):e0189610. doi: 10.1371/journal.pone.0189610 29281676 PMC5744948

[110] Schinske J, Tanner K. Teaching more by grading less (or differently). CBE—Life Sciences Education. 2014;13(2):159–66. doi: 10.1187/cbe.cbe-14-03-0054 26086649 PMC4041495

