# Supplementary material for: Breaking the mold: Study strategies of students who improve their achievement on introductory biology exams
Source: PLoS One. 2023 Jul 3;18(7):e0287313. doi: 10.1371/journal.pone.0287313 (PMC10317239; doi:10.1371/journal.pone.0287313)
Supplement: S1 Table — (PDF) [file pone.0287313.s001.pdf]

S1 Table. Students' self-reported demographic background information and school status (n = 376).

**Primary language**		**Intended first major**	
English	84.6%	Biology or neuroscience	38.3%
Bilingual or multilingual	13.8%		
Other than English	1.6%	Other STEM major (biomedical engineering, biochemistry, chemistry, psychology, environmental science, computer science)	28.2%
**Year at university**			
First year	85.1%	Applied health science majors§	16.8%
Second year	9.3%		
Third year	4.8%	Other/undecided	16.8%
Fourth year	0.8%		
**Concurrent chemistry enrollment**		**Pre-professional plans for health sciences**	
General Chemistry I with lab	79.0%	Medical school	64.9%
Not enrolled in chemistry	13.8%	Not planning to pursue careers in health sciences	17.8%
Basic Chemistry*	4.8%	Allied health (physical therapy, occupational therapy, or public health)	10.4%
Other chemistry course	2.4%	Dentistry, optometry, osteopathy, podiatry, or veterinary medicine	6.9%

*Basic Chemistry is a course for students who do not have sufficient background or mathematical skills necessary for General Chemistry I; it is taken as a preparatory class before General Chemistry I.
§These include health sciences, investigative medical sciences, nutrition/dietetics, clinical laboratory science, occupational therapy, physical therapy, athletic training, radiation therapy, magnetic resonance imaging, and emergency management.
