# Supplementary material for: Breaking the mold: Study strategies of students who improve their achievement on introductory biology exams
Source: PLoS One. 2023 Jul 3;18(7):e0287313. doi: 10.1371/journal.pone.0287313 (PMC10317239; doi:10.1371/journal.pone.0287313)
Supplement: S2 Table — Strategies are listed from most to least used, based on the overall sample’s responses. The χ² value from the contingency analysis is reported for each strategy (df = 3; adjusted α = 0.002941). Strategies having a significant association with exam score are highlighted, and boldfaced χ² values indicate statistical significance (p < 0.0001 for reviewing graded work, self-evaluation, using practice exams, monitoring understanding; p < 0.001 for reviewing textbook/screencasts, seeking instructor assistance. The p-values for the remaining strategies range from 0.0222 (goal-setting and planning) to 0.834 (self-consequating). (PDF) [file pone.0287313.s002.pdf]

S2 Table. Frequency with which students who earned different scores on Exam 1 (based on z-score groupings) reported higher use (Often/Very often) of each of the 17 SRL study strategies on the SRL1 survey.

	Exam 1 z-score group (n)					All students (345)
**SRL Strategy**	**Group 1 (52)**	**Group 2 (89)**	**Group 3 (136)**	**Group 4 (68)**	χ^2	
Reviewing notes	86.5%	92.1%	94.1%	97.1%	5.449	93.0%
Seeking information	86.5%	86.5%	93.4%	98.5%	9.519	91.6%
Keeping records	82.7%	93.3%	91.2%	97.1%	8.341	91.6%
Reviewing graded work	59.6%	75.3%	87.5%	97.1%	**33.652**	82.0%
Reviewing textbook/screencasts	71.2%	70.8%	89.7%	89.7%	**19.958**	82.0%
Self-evaluation	65.4%	73.0%	86.8%	94.1%	**22.819**	81.4%
Using practice exams	59.6%	77.5%	88.2%	89.7%	**24.525**	81.4%
Environmental structuring	71.2%	82.0%	77.9%	85.3%	4.160	79.4%
Monitoring understanding	53.8%	60.7%	73.5%	92.6%	**27.947**	71.0%
Seeking assistance from peers	61.5%	68.5%	70.6%	66.2%	1.523	67.8%
Rehearsing and memorizing	71.2%	61.8%	64.7%	61.8%	1.508	64.3%
Organizing and transforming	57.7%	46.1%	62.5%	66.2%	8.206	58.3%
Goal-setting and planning	38.5%	43.8%	57.4%	60.3%	9.611	51.6%
Studying with peers	28.8%	33.7%	28.7%	35.3%	1.325	31.3%
Seeking assistance from other resources	21.2%	32.6%	34.6%	30.9%	3.235	31.3%
Self-consequating	30.8%	28.1%	26.5%	23.5%	0.864	27.0%
Seeking instructor assistance	5.8%	12.4%	23.5%	29.4%	**14.985**	19.1%

Strategies are listed from most to least used, based on the overall sample's responses. The χ^2 value from the contingency analysis is reported for each strategy ($df = 3$; adjusted $\alpha = 0.002941$). Strategies having a significant association with exam score are highlighted, and boldfaced χ^2 values indicate statistical significance ($p < 0.0001$ for *reviewing graded work, self-evaluation, using practice exams, monitoring understanding*; $p < 0.001$ for *reviewing textbook/screencasts, seeking instructor assistance*. The *p*-values for the remaining strategies range from 0.0222 (*goal-setting and planning*) to 0.834 (*self-consequating*).
