# Supplementary material for: Breaking the mold: Study strategies of students who improve their achievement on introductory biology exams
Source: PLoS One. 2023 Jul 3;18(7):e0287313. doi: 10.1371/journal.pone.0287313 (PMC10317239; doi:10.1371/journal.pone.0287313)
Supplement: S3 Table — Bonferroni correction applied for six pairwise tests per strategy factor; adjusted α = 0.008333. (PDF) [file pone.0287313.s003.pdf]

S3 Table. Post-hoc Mann-Whitney U test results for pairwise comparisons, strategy factor scores by Exam 1 z-score groups.

Pairwise comparison	Housekeeping strategies	Use of course materials	Metacognitive strategies
**Group 1-Group 2**	$U = 2358.5$, $p = 0.8492$	$U = 1958.5$, $p = 0.1246$	$U = 1794$, $p = 0.025$
**Group 1-Group 3**	W = 2911, $p = 0.05785$	$U = 2080.5$, $p < 0.0001$	W = 1851, $p < 0.0001$
**Group 1-Group 4**	$U = 1423.5$, $p = 0.06559$	$U = 943$, $p < 0.0001$	$U = 524$, $p < 0.0001$
**Group 2-Group 3**	$U = 5009.5$, $p = 0.02735$	$U = 4080.5$, $p < 0.0001$	$U = 4142.5$, $p < 0.0001$
**Group 2-Group 4**	$U = 2459$, $p = 0.04299$	$U = 1834$, $p < 0.0001$	$U = 1266$, $p < 0.0001$
**Group 3-Group 4**	$U = 4494$, $p = 0.7419$	$U = 4334.5$, $p = 0.445$	$U = 3411$, $p = 0.002085$

Bonferroni correction applied for six pairwise tests per strategy factor; adjusted $\alpha = 0.008333$.
