# Supplementary material for: Breaking the mold: Study strategies of students who improve their achievement on introductory biology exams
Source: PLoS One. 2023 Jul 3;18(7):e0287313. doi: 10.1371/journal.pone.0287313 (PMC10317239; doi:10.1371/journal.pone.0287313)
Supplement: S1 File — (PDF) [file pone.0287313.s004.pdf]

S1 File. Self-regulated learning strategies survey, administered after Exam 1 (SRL1) and Exam 2 (SRL2).

For each of the following learning strategies, please mark how frequently you used them in preparing for Exam 1.
Very often = 5 Often = 4 Sometimes = 3 Rarely = 2 Never = 1

1. I evaluate the quality or progress of my work. For example, before turning in an assignment, I check over my work to make sure I did it right (I use a rubric if available, I make sure I answer questions to the best of my ability, etc.).	*Self-evaluation*
2. I review critically my graded assignments (homework, clicker questions, class worksheets); when I get an answer wrong, I try to understand what I missed and why.	*Reviewing graded work*
3. I rearrange and organize the information to improve my learning (for example, by making outlines, diagrams, summaries, study guides, etc.).	*Organizing and transforming*
4. I set goals and a timeline for studying the material and plan how to meet those goals on time (for example, I plan to review a chapter a day in the week before a test).	*Goal-setting and planning*
5. When I'm uncertain about an assignment, I look up the information I need to complete the assignment (either in my notes, in the course materials, or from other online or printed sources).	*Seeking information*
6. I take notes in class and/or when I study.	*Keeping records*
7. I monitor my understanding of the material and make a note of what I don't understand.	*Monitoring understanding*
8. I arrange my studying environment so I can learn more effectively (for example, I move to a quiet place or have background noise).	*Environmental structuring*
9. I plan to reward myself once I reach a learning goal; if I do not achieve my goal, I withhold the reward.	*Self-consequating*
10. When I study, I practice or rehearse important facts in order to memorize them (for example, I use flashcards, make acronyms, etc.).	*Rehearsing and memorizing*
11. I study with friends or classmates.	*Studying with peers*
12. If I don't understand something, I ask a friend or classmate for help.	*Seeking assistance from peers*
13. If I don't understand something, I ask the instructor for help or clarification (for example, I send an email, I attend office hours, or I approach the instructor before or after class).	*Seeking instructor assistance*
14. If I don't understand something, I ask a course LA, SI leader, or tutor for help.	*Seeking assistance from other resources*
15. To prepare for the exam, I reread my notes and/or the class slides.	*Reviewing notes*
16. To prepare for the exam, I practice answering previous years' exams.	*Using practice exams*
17. To prepare for the exam, I review the textbook readings and/or Tegrity® screencasts.	*Reviewing the textbook or screencasts*

18 [a]. Think about your study strategies, and whether you think they have worked well for you. If you wish to improve your outcome, perhaps you may want to consider trying a new approach. Either way, it is important to have a plan. Will you make any change in your study strategies to prepare for the next course exams? (1) No, I plan on studying just as I have done so far. (2) Yes, I plan on making minor changes to how I study. (3) Yes, I plan on substantially changing how I study.
If you answered "Yes," please briefly explain what you are changing.

Strategy names (italicized and shaded in the table, on the right) are based on Zimmerman and Martinez-Pons (1986, 1988) and were not shown to students during the survey; they are reported here as a reference and are used throughout the manuscript to report results. Both surveys were identical except where noted.
[a] See text below.

On the SRL2 survey, the study plan prompt was split into three separate questions:
18) You answered a similar questionnaire after Exam 1. At that time, you were invited to come up with a plan for how to study for the next course exams. Revisit your proposed study plan. To what extent did you follow your plan? (1= Not at all, 2 = Barely, 3 = Somewhat, 4 = Mostly, 5 = Completely)

19) How well do you think your plan worked for you? (1= Not at all, 2 = Barely, 3 = Somewhat, 4 = Mostly, 5 = Completely)

20) Now, make your plan for the rest of the semester. Will you make any change in your study strategies to prepare for the next course exams?

(1) No, I plan on studying just as I have done so far.
(2) Yes, I plan on making minor changes to how I study.
(3) Yes, I plan on substantially changing how I study.
If you answered "Yes," please briefly explain what you are changing.

Data from Question 18 on the SRL1 survey and Questions 18-20 on the SRL2 survey are not reported in the manuscript; the items are included here for reference since they were part of student-facing materials.

For this version of the SRL study strategies survey, used in the current study, we made six important changes compared to the original version in our previous work (Sebesta and Bray Speth 2017). First, we split the strategy "keeping records and monitoring" into "keeping records" and "monitoring understanding." We made this adjustment because students may not necessarily monitor their understanding when they take notes, and vice versa. Second, we added the strategy "studying with peers" to determine if students would generally study with friends and classmates versus seeking help specifically from friends and classmates, compared to other social sources (e.g., the instructor). Third, we moved the strategy "reviewing graded work" to follow immediately after "self-evaluation;" the former strategy was presented last in the initial version of the survey. We wished to make clear to students the distinction between evaluating work *before* versus *after* it was graded with feedback. Fourth, we changed the name of a strategy: item 16 is now called "using practice exams" instead of "reviewing exams." This name change better conveys the behavior in which students were engaging during their studying (using exams from prior semesters as a study resource, rather than reviewing exams they took for a grade in the course). Fifth, we adjusted the study plan prompt so students could answer on a Likert-type scale for the relative degree of change to their study plan, how much they followed the study plan they made after the first exam, and how well they believe their study plan worked. Adding Likert-type questions would allow for more straightforward analysis of their perceptions on study-plan follow-through and effectiveness. Sixth, we added in slightly more prompting in various items to help students identify examples of activities they may do that would align with given strategy (e.g., adding in ways to seek help from the instructor).
