# Supplementary material for: Breaking the mold: Study strategies of students who improve their achievement on introductory biology exams
Source: PLoS One. 2023 Jul 3;18(7):e0287313. doi: 10.1371/journal.pone.0287313 (PMC10317239; doi:10.1371/journal.pone.0287313)
Supplement: S3 File — (PDF) [file pone.0287313.s006.pdf]

S3 File. Means, standard error, and linear regression equation and statistical results for Exam 1 and the average of Exams 2/3/4 for three additional, consecutive cohorts.

Cohort (n)	Exam 1 % score mean ± SEM	Average Exams 2/3/4 % score mean ± SEM	Linear regression equation (R^2)	Regression statistical results
**Cohort 1 (n = 395)**	80.70 ± 0.70	75.45 ± 0.73	*Avg Ex 2/3/4 % score* = 4.116 + 0.884(*Ex 1 % score*) (R^2 = 0.716)	*F*(1, 393) = 988.7; $p < 0.0001$
**Cohort 2 (n = 260)**	73.71 ± 0.95	74.50 ± 0.93	*Avg Ex 2/3/4 % score* = 13.255 + 0.831(*Ex 1 % score*) (R^2 = 0.723)	*F*(1, 258) = 674.7; $p < 0.0001$
**Cohort 3 (n = 223)**	74.62 ± 1.11	76.32 ± 1.03	*Avg Ex 2/3/4 % score* = 16.722 + 0.799(*Ex 1 % score*) (R^2 = 0.742)	*F*(1, 221) = 636.6; $p < 0.0001$

For each cohort, we satisfied two assumptions for a univariate linear regression: the exam percent scores were significantly correlated with one another (Cohort 1: Spearman's rho = 0.844, *S* = 1600644, $p < 0.0001$; Cohort 2: Spearman's rho = 0.833, *S* = 488636, $p < 0.0001$; Cohort 3: Spearman's rho = 0.859, *S* = 259969, $p < 0.0001$), and model residuals had a mean of zero. According to Shapiro-Wilk tests, model residuals were normally distributed only for Cohort 2 ($W = 0.99787$, $p = 0.9839$) but not for the other cohorts (Cohort 1: $W = 0.97305$, $p < 0.0001$; Cohort 3: $W = 0.98702$, $p = 0.04023$). Although neither exam variable was normally distributed for any cohort, based on Shapiro-Wilk tests (Cohort 1: Exam 1 $W = 0.95076$, $p < 0.0001$; Avg. Exams 2/3/4 $W = 0.95715$, $p < 0.0001$. Cohort 2: Exam 1 $W = 0.95715$, $p < 0.0001$; Avg. Exams 2/3/4 $W = 0.95134$, $p < 0.0001$. Cohort 3: Exam 1 $W = 0.94685$, $p < 0.0001$; Avg. Exams 2/3/4 $W = 0.94368$, $p < 0.0001$), they had nearly identical distribution shapes that allowed for fairer comparison, so we proceeded with interpreting linear regression models.

For each cohort, we found a strong, positive predictive relationship between students' Exam 1 percent score and the average percent score of their Exams 2, 3, and 4 (see also S2 Fig), just as we found for the cohort reported in the main text. Exam 1 alone accounted for approximately 72% of the variance in the Exams 2/3/4 for Cohort 1 (R^2 = 0.716) and Cohort 2 (R^2 = 0.723) and approximately 74% for Cohort 3 (R^2 = 0.742).
