# Supplementary figures and images for: Breaking the mold: Study strategies of students who improve their achievement on introductory biology exams
Source: PLoS One. 2023 Jul 3;18(7):e0287313. doi: 10.1371/journal.pone.0287313 (PMC10317239; doi:10.1371/journal.pone.0287313)

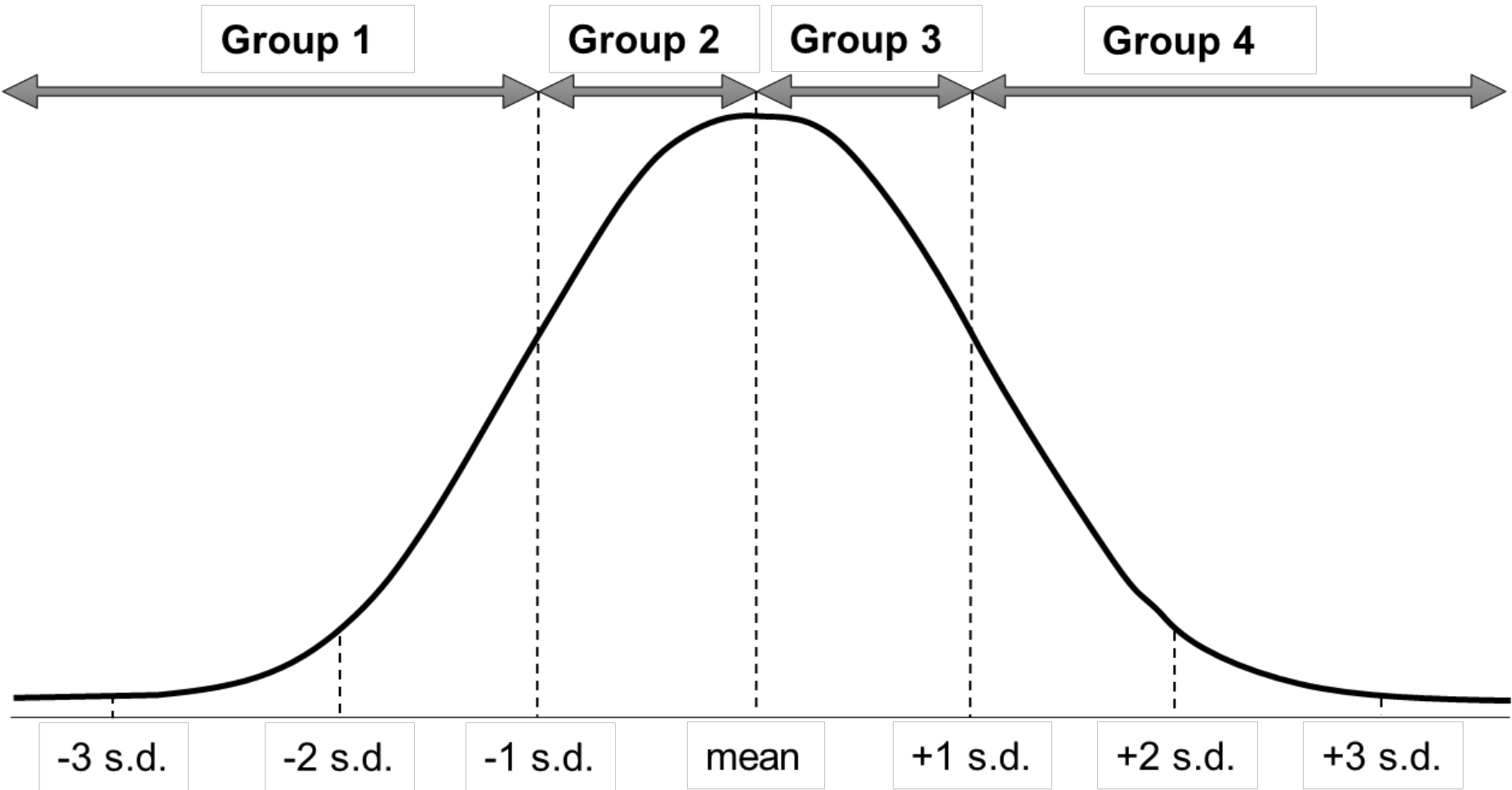

Supplement: S1 Fig — (PDF) [file pone.0287313.s008.pdf]
