# Supplementary material for: Breaking the mold: Study strategies of students who improve their achievement on introductory biology exams
Source: PLoS One. 2023 Jul 3;18(7):e0287313. doi: 10.1371/journal.pone.0287313 (PMC10317239; doi:10.1371/journal.pone.0287313)
Supplement: S2 Fig — (A) Linear regression for Year 1 cohort, (B) linear regression for Year 2 cohort, and (C) linear regression for Year 3 cohort. The equation and R2 value for each cohort are also provided, as well as the 95% confidence interval for the regression line. (PDF) [file pone.0287313.s009.pdf]

(A) Year 1 cohort (n = 395)

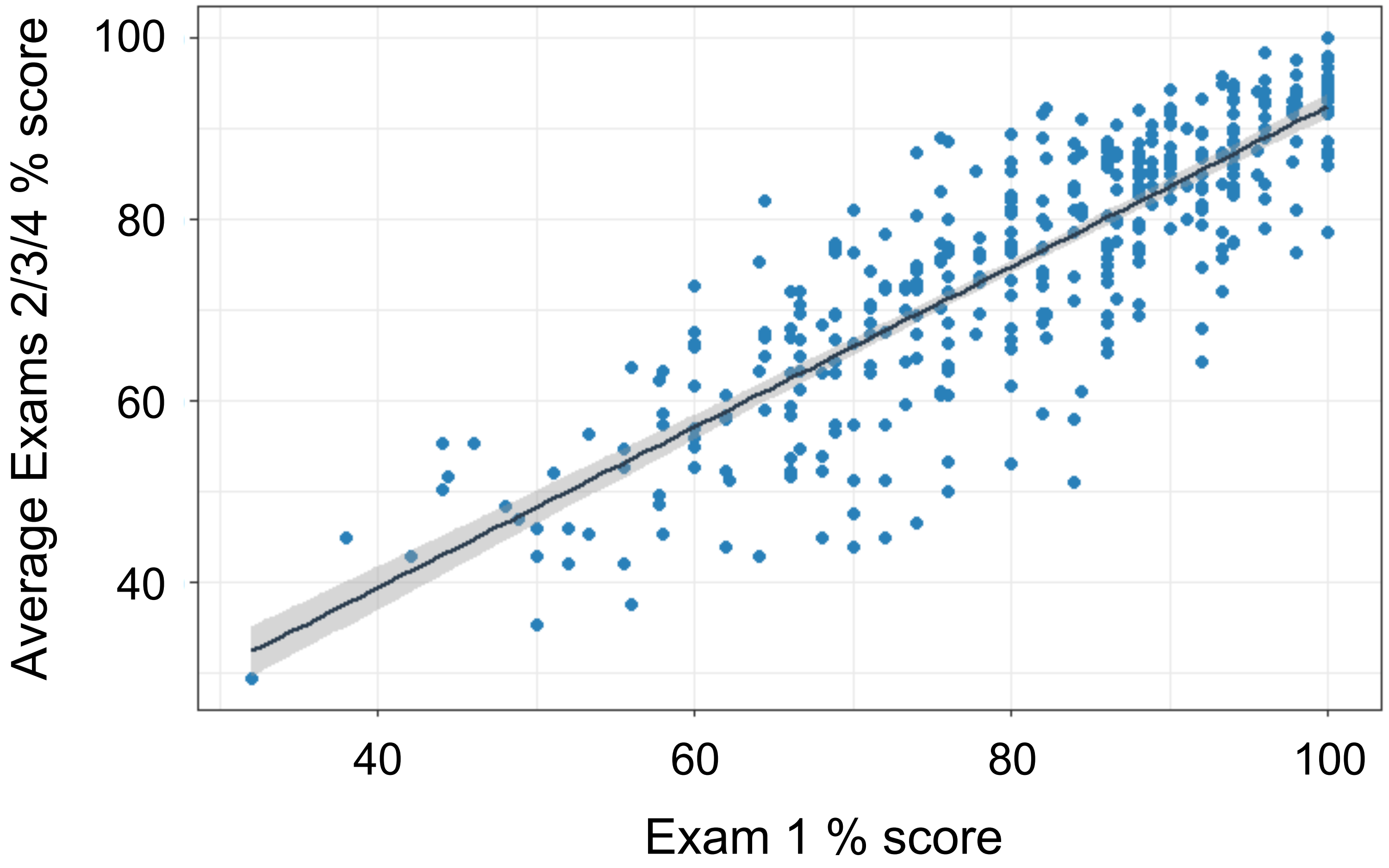

Avg Ex 2/3/4 % score = 4.116 + 0.884(*Ex 1 % score*)
(R^2 = 0.716)

(B) Year 2 cohort (n = 260)

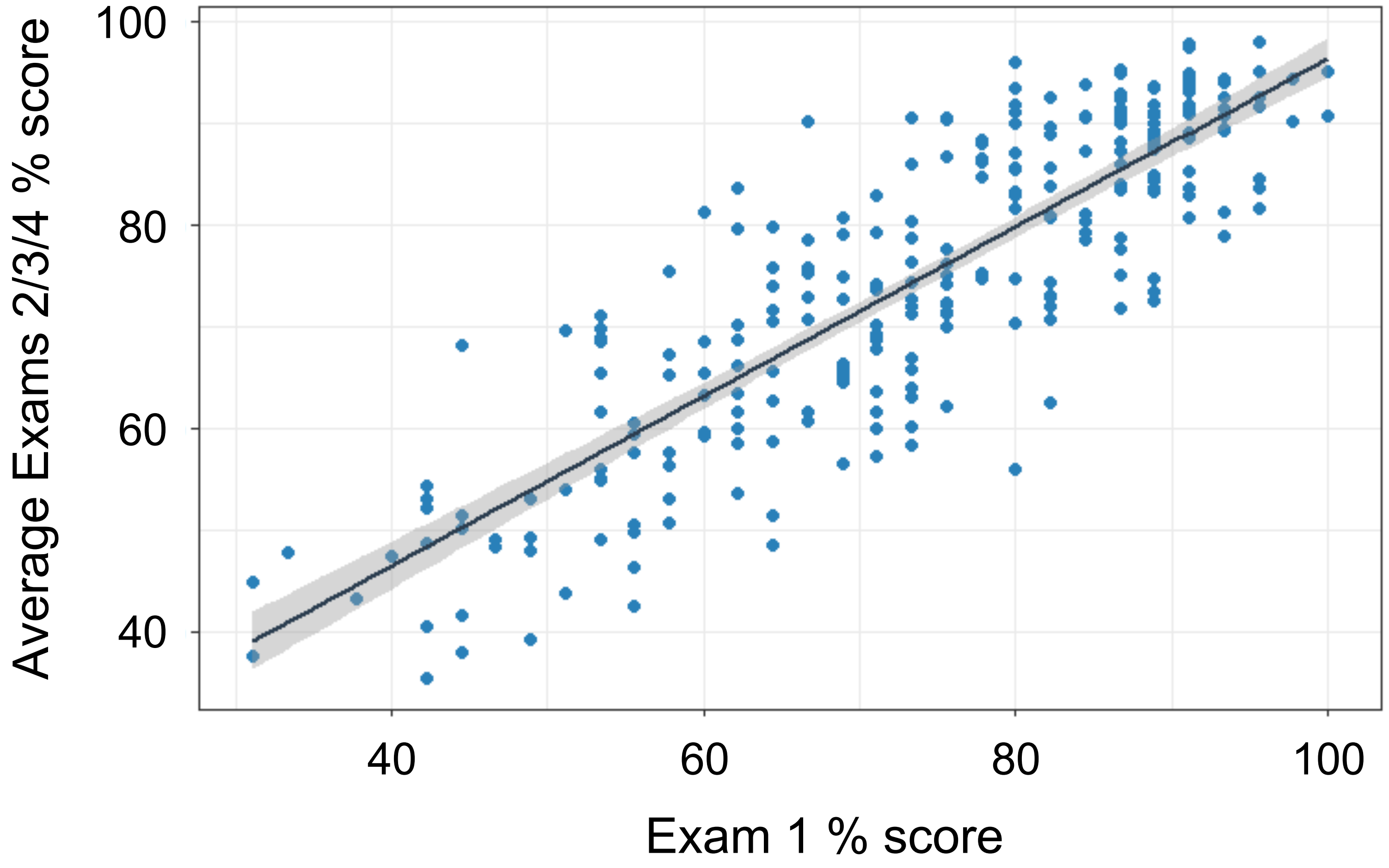

Avg Ex 2/3/4 % score = 13.255 + 0.831(*Ex 1 % score*)
(R^2 = 0.723)

(C) Year 3 cohort (n = 223)

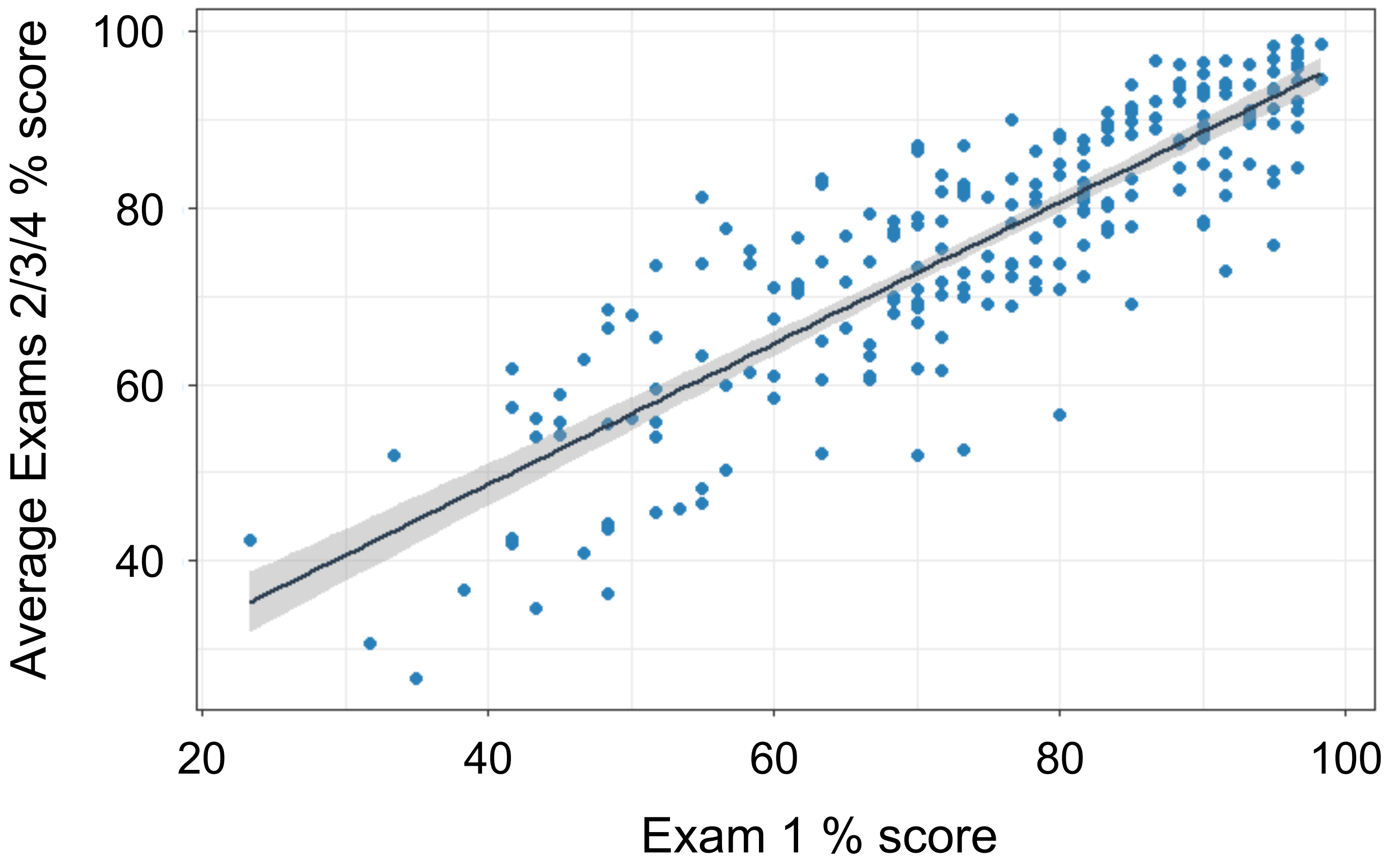

Avg Ex 2/3/4 % score = 16.722 + 0.799(*Ex 1 % score*)
(R^2 = 0.742)
