# Supplementary material for: Breaking the mold: Study strategies of students who improve their achievement on introductory biology exams
Source: PLoS One. 2023 Jul 3;18(7):e0287313. doi: 10.1371/journal.pone.0287313 (PMC10317239; doi:10.1371/journal.pone.0287313)
Supplement: S3 Fig — The outlined boxes represent the learning phases in Hattie and Donoghue’s model. Light blue-shaded boxes represents the factors emerging from our EFA, each listing the study strategies that loaded onto each factor. (PDF) [file pone.0287313.s010.pdf]

Strategy factors mapped onto the phases of learning (Hattie & Donoghue 2016)

Surface learning
Acquiring & consolidating knowledge base

Deep learning
Monitoring and refining understanding, connecting ideas

Transfer
Applying knowledge and understanding to novel contexts

Factor 1: Housekeeping strategies

-Organizing and transforming
-Rehearsing and memorizing
-Goal-setting and planning

Factor 2: Use of course materials

-Reviewing notes
-Reviewing textbook/screencasts
-Using practice exams

Factor 3: Metacognitive strategies

-Self-evaluation
-Reviewing graded work
-Monitoring understanding
-Seeking instructor assistance
